# A modifiable risk factors atlas of lung cancer: A Mendelian randomization study

**DOI:** 10.1002/cam4.4015

**Published:** 2021-06-02

**Authors:** Jiayi Shen, Huaqiang Zhou, Jiaqing Liu, Yaxiong Zhang, Ting Zhou, Yunpeng Yang, Wenfeng Fang, Yan Huang, Li Zhang

**Affiliations:** ^1^ Department of Medical Oncology Sun Yat‐sen University Cancer Center State Key Laboratory of Oncology in South China Collaborative Innovation Center for Cancer Medicine Guangzhou China; ^2^ Zhongshan School of Medicine Sun Yat‐sen University Guangzhou China

**Keywords:** causality, lung cancer, Mendelian randomization, risk factor

## Abstract

**Background:**

There has been no study systematically assessing the causal effects of putative modifiable risk factors on lung cancer. In this study, we aimed to construct a modifiable risk factors atlas of lung cancer by using the two‐sample Mendelian randomization framework.

**Methods:**

We included 46 modifiable risk factors identified in previous studies. Traits with *p*‐value smaller than 0.05 were considered as suggestive risk factors. While the Bonferroni corrected *p*‐value for significant risk factors was set to be 8.33 × 10^−4^.

**Results:**

In this two‐sample Mendelian randomization analysis, we found that higher socioeconomic status was significantly correlated with lower risk of lung cancer, including years of schooling, college or university degree, and household income. While cigarettes smoked per day, time spent watching TV, polyunsaturated fatty acids, docosapentaenoic acid, eicosapentaenoic acid, and arachidonic acid in blood were significantly associated with higher risk of lung cancer. Suggestive risk factors for lung cancer were found to be serum vitamin A1, copper in blood, docosahexaenoic acid in blood, and body fat percentage.

**Conclusions:**

This study provided the first Mendelian randomization assessment of the causality between previously reported risk factors and lung cancer risk. Several modifiable targets, concerning socioeconomic status, lifestyle, dietary, and obesity, should be taken into consideration for the development of primary prevention strategies for lung cancer.

## Graphical abstract

With the utilization of Mendelian randomization analysis, we provided the evidence for the relationship between previously reported risk factors and lung cancer from the aspect of causation. We identified several modifiable targets for primary prevention of lung cancer, concerning socioeconomic status, lifestyle, dietary and obesity.

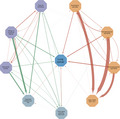

## 1 BACKGROUND

Lung cancer is the most commonly diagnosed cancer and the leading cause of cancer‐related death in the world.^1^ It accounted for around 2 million new cases and 1.8 million deaths in 2018.^1^ Nearly half of the lung cancer patients present with advanced disease at the time of initial diagnosis due to the lack of specific signs or symptoms.^2^ Lung cancer is typically associated with a poor prognosis, and its overall 5‐year survival rate is less than 20%. Although there is a reduction in incidence and mortality along with treatment advances in the past decades, lung cancer remains to be an immense disease and economic burden.^3^ Given the limited survival benefit from comprehensive anticancer therapy, it is important to better understand the etiology of lung cancer and establish proper primary prevention strategies for disease control.

According to the Cancer Statistics report from American Cancer Society, about 60% of cancers can be avoided by reducing exposure to risk factors.^4^ For example, smoking is an established cause of lung cancer. National Tobacco Control Programs have effectively reduced the incidence and mortality of lung cancer in the United States. Despite the control of tobacco consumption, lung cancer incidence is still high.^1^ There were also lung cancer patients who were not exposed to tobacco.^5^ In regard to the high incidence of lung cancer and the unknown etiologies, there has been an increasing interest in the development of comprehensive lung cancer prevention strategies by identifying and reducing exposure to risk factors of lung cancer.

The World Cancer Research Fund (WCRF) and the American Institute for Cancer Research (AICR) have indicated that there is strong evidence that smoking is an established cause of lung cancer, and that arsenic in drinking water and beta‐carotene supplements increase the risk of lung cancer.^6^ They also concluded that evidence is too limited to establish causal associations for many other modifiable risk factors concerning diet and nutrition.^6^ In general, a few risk factors have been linked to lung cancer in observational epidemiological studies with conclusive evidence.^7^ However, retrospective observational studies are usually susceptible to residual confounding bias and reverse causation. Moreover, data from prospective randomized trials are scarce and sometimes infeasible in practice.^8^

Mendelian randomization (MR) is a novel analytical approach that uses genetic variants as instrumental variables (IVs) to assess causal inference between risk factors and outcomes.^9^ The principle of MR is that the alleles of genetic variants are randomly allocated at gamete formation, a process somewhat similar to the random assignment of participants in a randomized controlled trial.^10^ The MR design will not be vulnerable to reverse causation and generally free of confounders, which are common in conventional observational studies.^10^ In addition, we can implement the MR approach using the published summary data from 2 independent large‐scale genome‐wide association studies (GWAS), which greatly increases the scope and statistical power of MR.^11^, ^12^

To date, we have recently used MR to examine the relationship between lung cancer and education, polyunsaturated fatty acid, minerals et al.^13^, ^14^, ^15^, ^16^, ^17^ However, there has been no study systematically assessing the causal effects of potentially modifiable risk factors on lung cancer. Here, we have extended our analysis to examine 46 potentially modifiable risk factors for lung cancer using a two‐sample MR framework.

## 2 METHODS

### 2.1 Identification of putative modifiable risk factors of lung cancer

**FIGURE 1:**
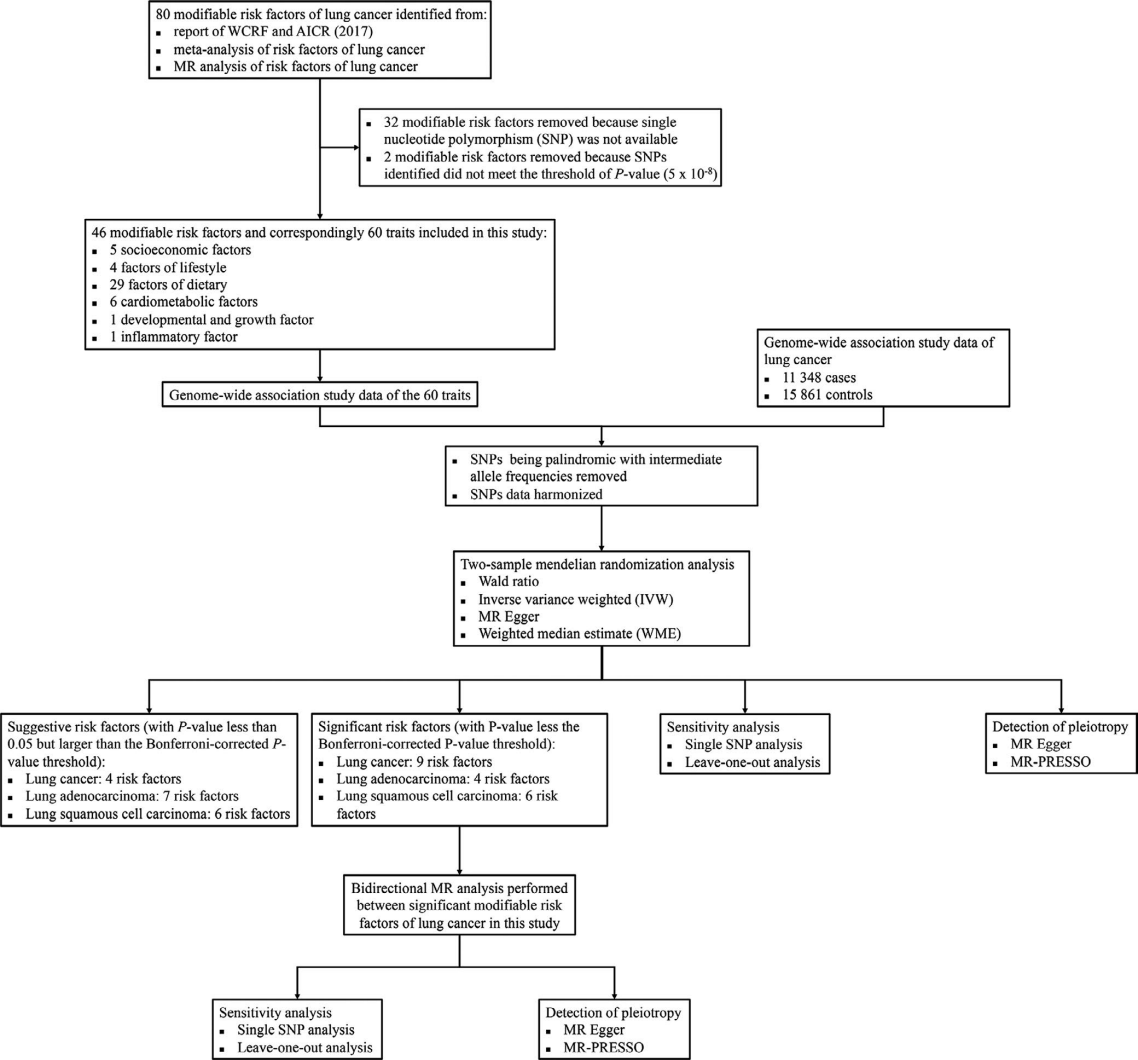
Flow chart. Study design of this MR analysis

We identified 80 putative modifiable risk factors of lung cancer from three sources: (a) a report about the relationship between diet, nutrition, physical activity, and lung cancer by WCRF/AICR^6^; (b) published meta‐analysis about risk factors of lung cancer; (c) published MR analysis about risk factors of lung cancer (Figure 1). To identify epidemiological meta‐analyses focusing on the modifiable risk factors of lung cancer, we searched PubMed with the terms: ‘((lung cancer) AND risk factor) AND meta‐analysis. The date of publication was restricted from the previous 10 years (searching conducted on 23 March 2020). Mendelian randomization analyses of risk factors of lung cancer were collected by searching PubMed with the terms: ‘(lung cancer) AND ((Mendelian randomization) OR Mendelian randomisation)’ (searching conducted on 23 March 2020). We precluded 34 identified risk factors, because genetic IVs that satisfied our criterion were not available. Sources and inclusion of the identified risk factors were detailed in Table S1. We retained 46 putative modifiable risk factors of lung cancer.

### 2.2 Genome‐wide association study data of risk factors of lung cancer

We searched Pubmed and MR base for GWAS data of the identified putative risk factors of lung cancer. Source of GWAS for each trait was identified in Table 1. Threshold of *p*‐value for the association between single nucleotide polymorphisms (SNPs) and traits was set to be 5 × 10^−8^. In the situation when R^2^ was not provided by the GWAS, we calculated it using data from MRbase.^18^ Power and F‐statistic were calculated with four assumed odds ratios (ORs).^19^ R^2^ of the SNPs, power and F‐statistic were shown in Table 2 for lung cancer and Table S2 for lung adenocarcinoma (LUAD) and lung squamous cell carcinoma (LUSC). But we did not filter the SNPs according to the calculated F‐statistic, because it was promoted by Stephen Burgess et al. that the selection of IVs according to F‐statistic can introduce additional biases.^20^ We treated R^2^, power and F‐statistic as the characteristics of the GWAS of traits, which was useful in the sensitivity analysis for weak instrument bias. Finally, we got 60 traits corresponding to the 46 included risk factors, because some risk factors had more than 1 traits (Table S2). To avoid bias caused by linkage disequilibrium, we selected the SNPs that achieved independence at linkage disequilibrium (LD) r2 = 0.001 and a distance of 10,000 kb. Effect of each SNP on its corresponding trait was also shown in Table S3.

**TABLE 1:** Source and number of SNPs of GWAS data of exposure used in this MR analysis

Trait	Source	Number of SNPs available	Number of SNPs used
Socioeconomic			
Years of schooling	10.1038/nature17671	73	73
College or university degree	www.mrbase.org, Consortium: MRC‐IEU, First author: Ben Elsworth.	261	250
Unemployed	www.mrbase.org, Consortium: MRC‐IEU, First author: Ben Elsworth.	2	1
In paid employment or self‐employed	www.mrbase.org, Consortium: MRC‐IEU, First author: Ben Elsworth.	1	1
Household income	www.mrbase.org, Consortium: MRC‐IEU, First author: Ben Elsworth.	48	45
Townsend deprivation index	www.mrbase.org, Consortium: MRC‐IEU, First author: Ben Elsworth.	18	17
Lifestyle			
Cigarettes smoked per day	10.1038/ng.571	1	1
Accelerometer‐based physical activity	10.1038/s41467‐020–14389–8	5	4
Time spent watching television	www.mrbase.org, Consortium: MRC‐IEU, First author: Ben Elsworth.	113	108
Sedentary behaviors	10.1038/s41467‐018–07743–4	4	4
Dietary			
Bowls of cereal per week	10.1038/s41467‐020–15193–0	21	14
Tablespoons of cooked vegetables per day	10.1038/s41467‐020–15193–0	11	7
Tablespoons of raw vegetables per day	10.1038/s41467‐020–15193–0	11	9
Pieces of dried fruit per day	10.1038/s41467‐020–15193–0	11	10
Pieces of fresh fruit per day	10.1038/s41467‐020–15193–0	45	38
Overall beef intake	10.1038/s41467‐020–15193–0	5	2
Overall lamb/mutton intake	10.1038/s41467‐020–15193–0	9	8
Overall pork intake	10.1038/s41467‐020–15193–0	7	5
Processed meat intake	10.1038/s41467‐020–15193–0	8	7
Poultry intake	10.1038/s41467‐020–15193–0	3	3
Overall non‐oily fish intake	10.1038/s41467‐020–15193–0	2	2
Overall oily fish intake	10.1038/s41467‐020–15193–0	37	27
Never eat eggs versus no eggs restrictions	10.1038/s41467‐020–15193–0	1	1
Overall alcohol intake	10.1038/s41467‐020–15193–0	29	21
Cups of coffee per day	10.1038/s41467‐020–15193–0	23	15
Cups of tea per day	10.1038/s41467‐020–15193–0	29	21
Carbohydrate intake	10.1038/s41380‐020–0697–5	13	2
Protein intake	10.1038/s41380‐020–0697–5	7	7
Fat intake	10.1038/s41380‐020–0697–5	6	6
Serum vitamin A1 (Retinol)	10.1093/hmg/ddr387	2	2
Vitamin B6 blood concentration	10.1016/j.ajhg.2009.02.011	1	1
Serum vitamin B12	10.1371/journal.pgen.1003530	9	8
Circulating hydroxyvitamin D	10.1038/s41467‐017–02662–2	5	5
Serum vitamin E	10.1093/hmg/ddr296	3	2
Homocysteine blood concentration	10.1016/j.ajhg.2009.02.011	1	1
Circulating carotenoids	10.1016/j.ajhg.2008.12.019	1	1
Inorganic arsenic in urine (%) (iAs%)	10.1093/ije/dyz046	3	2
Monomethylarsenate in urine (%) (MMA%)	10.1093/ije/dyz046	3	2
Dimethylarsinate in urine (%) (DMA%)	10.1093/ije/dyz046	3	2
Serum calcium	10.1371/journal.pgen.1003796	7	7
Cooper in blood	10.1093/hmg/ddt239	2	2
Biochemical markers for iron status (serum iron, transferrin, transferrin saturation and ferritin)	10.1038/ncomms5926	3	3
Selenium in blood	10.1093/hmg/ddt239	1	1
Zinc in blood	10.1093/hmg/ddt239	2	2
Other polyunsaturated fatty acids than 18:2 in blood	10.1038/ncomms11122	11	10
Docosahexaenoic acid (DHA) (22:6n‐3) in blood	10.1038/ncomms11122	6	5
Docosapentaenoic acid (DPA) (22:5n‐3) in blood	10.1038/ng.2982	1	1
Eicosapentaenoic acid (EPA) (20:5n‐3) in blood	10.1038/ng.2982	1	1
Arachidonic acid (AA) (20:4n‐6) in blood	10.1038/ng.2982	1	1
Dihomo‐γ‐linolenic acid (DGLA) (20:3n‐6) in blood	10.1038/ng.2982	2	2
Linoleic acid (LA) (18:2n‐6) in blood	10.1038/ncomms11122	16	15
Low‐density lipoprotein cholesterol level in blood	10.1038/ng.2797	80	76
Cardiometabolic			
Body mass index	10.1038/nature14177	79	79
Body fat percentage	www.mrbase.org, Consortium: MRC‐IEU, First author: Ben Elsworth.	394	376
Waist circumference	10.1038/nature14132	42	42
Waist to hip ratio	10.1038/nature14132	38	37
Circulating adiponectin	10.1371/journal.pgen.1002607	14	14
Fasting insulin interaction with body mass index	10.1038/ng.2274	10	6
Developmental and growth factors			
Adult height	10.1038/ng.3097	386	382
Inflammatory			
Serum C‐reactive protein	10.1093/hmg/ddq551	3	3

**TABLE 2:** R^2^, power, and F‐statistics of GWAS data of exposure used in the MR analysis between risk factors and lung cancer

Trait	R^2 a^	Power to identify OR_SD_ ^b^ of				F‐statistic
		0.91 or 1.10	0.83 or 1.20	0.75 or 1.33	0.67 or 1.50	
Socioeconomic						
Years of schooling	0.0043	0.08	0.17	0.32	0.62	118.50
College or university degree	0.0255^c^	0.24	0.67	0.96	1.00	711.97
Unemployed	0.0001^c^	0.05	0.05	0.05	0.06	3.23
In paid employment or self‐employed	0.0001^c^	0.05	0.05	0.05	0.06	2.90
Household income	0.0046^c^	0.08	0.18	0.34	0.65	127.41
Townsend deprivation index	0.0013^c^	0.06	0.08	0.13	0.23	35.64
Lifestyle						
Cigarettes smoked per day	0.0050	0.09	0.19	0.37	0.68	137.73
Accelerometer‐based physical activity	0.0020	0.06	0.10	0.18	0.34	55.53
Time spent watching television	0.0102^c^	0.12	0.33	0.64	0.93	281.06
Sedentary behaviors	0.0008	0.06	0.07	0.10	0.16	22.78
Dietary						
Bowls of cereal per week	NA^d^	NA	NA	NA	NA	NA
Tablespoons of cooked vegetables per day	NA^d^	NA	NA	NA	NA	NA
Tablespoons of raw vegetables per day	NA^d^	NA	NA	NA	NA	NA
Pieces of dried fruit per day	NA^d^	NA	NA	NA	NA	NA
Pieces of fresh fruit per day	NA^d^	NA	NA	NA	NA	NA
Overall beef intake	NA^d^	NA	NA	NA	NA	NA
Overall lamb/mutton intake	NA^d^	NA	NA	NA	NA	NA
Overall pork intake	NA^d^	NA	NA	NA	NA	NA
Processed meat intake	NA^d^	NA	NA	NA	NA	NA
Poultry intake	NA^d^	NA	NA	NA	NA	NA
Overall non‐oily fish intake	NAd	NA	NA	NA	NA	NA
Overall oily fish intake	NA^d^	NA	NA	NA	NA	NA
Never eat eggs vs. no eggs restrictions	NA^d^	NA	NA	NA	NA	NA
Overall alcohol intake	NA^d^	NA	NA	NA	NA	NA
Cups of coffee per day	NA^d^	NA	NA	NA	NA	NA
Cups of tea per day	NA^d^	NA	NA	NA	NA	NA
Carbohydrate intake	0.00011–0.00027^e^	NA	NA	NA	NA	NA
Protein intake	0.00015–0.00050^e^	NA	NA	NA	NA	NA
Fat intake	0.00012–0.00054^e^	NA	NA	NA	NA	NA
Serum vitamin A1 (Retinol)	0.0070	0.10	0.24	0.48	0.82	192.81
Vitamin B6 blood concentration	0.0140	0.15	0.43	0.77	0.98	387.33
Serum vitamin B12	0.0470	0.40	0.90	1.00	1.00	1342.89
Circulating hydroxyvitamin D	0.0265	0.25	0.69	0.96	1.00	741.67
Serum vitamin E	0.0065	0.10	0.23	0.46	0.79	179.02
Homocysteine blood concentration	NA^d^	NA	NA	NA	NA	NA
Circulating carotenoids	0.0277	0.26	0.71	0.97	1.00	776.16
Inorganic arsenic in urine (%) (iAs%)	NA^d^	NA	NA	NA	NA	NA
Monomethylarsenate in urine (%) (MMA%)	NA^d^	NA	NA	NA	NA	NA
Dimethylarsinate in urine (%) (DMA%)	NA^d^	NA	NA	NA	NA	NA
Serum calcium	0.0258	0.24	0.68	0.96	1.00	721.58
Cooper in blood	0.0500	0.42	0.92	1.00	1.00	1433.05
Biochemical markers for iron status (serum iron, transferrin, transferrin saturation and ferritin)	0.0115	0.13	0.37	0.69	0.96	317.54
Selenium in blood	0.0400	0.35	0.85	1.00	1.00	1134.71
Zinc in blood	0.0800	0.60	0.99	1.00	1.00	2367.00
Other polyunsaturated fatty acids than 18:2 in blood	0.1100^c^	0.74	1.00	1.00	1.00	3363.31
Docosahexaenoic acid (DHA) (22:6n‐3) in blood	0.0194^c^	0.19	0.56	0.89	1.00	538.71
Docosapentaenoic acid (DPA) (22:5n‐3) in blood	0.0171^c^	0.18	0.50	0.85	0.99	473.12
Eicosapentaenoic acid (EPA) (20:5n‐3) in blood	0.0121^c^	0.14	0.38	0.71	0.97	333.62
Arachidonic acid (AA) (20:4n‐6) in blood	0.0474^c^	0.40	0.91	1.00	1.00	1355.97
Dihomo‐γ‐linolenic acid (DGLA) (20:3n‐6) in blood	0.0172^c^	0.18	0.51	0.85	0.99	476.65
Linoleic acid (LA) (18:2n‐6) in blood	0.0690^c^	0.54	0.98	1.00	1.00	2017.66
Low‐density lipoprotein cholesterol level in blood	0.1460	0.85	1.00	1.00	1.00	4652.66
Cardiometabolic						
Body mass index	0.0270	0.25	0.70	0.96	1.00	756.03
Body fat percentage	0.0486^c^	0.41	0.91	1.00	1.00	1391.14
Waist circumference	0.0108^c^	0.13	0.35	0.66	0.95	297.50
Waist to hip ratio	0.0140	0.15	0.43	0.77	0.98	387.33
Circulating adiponectin	0.0178	0.18	0.52	0.86	1.00	494.10
Fasting insulin interaction with body mass index	0.0060^c^	0.09	0.21	0.43	0.76	164.95
Developmental and growth factors						
Adult height	0.1600	0.88	1.00	1.00	1.00	5183.67
Inflammatory						
Serum C‐reactive protein	0.0140	0.15	0.43	0.77	0.98	387.33

^a^ R^2^ (Proportion of variance explained by SNPs).

^b^ OR_SD_ for the estimation of associations between exposures and lung cancer.

^c^ R^2^ of these exposures was calculated using data from MRbase, because information of these genetic variants was not published or R^2^ was not included in the article.

^d^ R^2^ of these exposures was not available, because R^2^ was not included in the article.

^e^ R^2^ of all the SNPs utilized was not available. Thus the minimal R^2^ and the maximal R^2^ of the individual SNPs utilized were provided.

### 2.3 GWAS data of lung cancer

GWAS data of lung cancer, LUAD, and LUSC were derived from a meta‐analysis of four previously reported GWASs by the International Lung Cancer Consortium.^21^ The four GWASs were all based on European population and comprised 11,348 cases of lung cancer and 15,861 controls, 3,442 cases of LUAD and 14,894 controls, and 3,275 cases of LUSC and 15,038 controls. SNPs were genotyped making use of Illumina HumanHap 317, 317+240S, 370, 550, 610 or 1 M arrays. All of the GWASs were reviewed and approved by the ethics committees in the original source articles.

### 2.4 Study design: Two‐sample MR analysis

We used an MR approach to investigate the association between different risk factors and risk of lung cancer. MR study is a novel epidemiological method for the evaluation of the causation between exposure and outcome, utilizing genetic IVs (SNPs) of exposure as proxies. The MR method was based on the following three key assumptions: (A) The IVs is associated with the risk factor (Relevance); (B) The IVs affects the outcome only through the risk factor (Exclusion restriction); and (C) The IVs is not associated with any confounders (Independent).^22^ Assumptions of MR study and study design are shown in Figure S1. To estimate a causal effect with IV analysis, additional assumptions are required. The associations are linear and not affected by statistical interactions.^23^ Two‐sample MR is an extension in which the effects of the genetic instrument on the exposure and on the outcome are obtained from the published summary data of separate GWAS, which greatly increases the scope of MR.

### 2.5 Statistical method

Wald ratio estimate was performed if there was only 1 SNP for the trait, in which SNP‐outcome association was divided by its SNP‐exposure association to obtain the causal relationship.^24^ Inverse variance weighted (IVW) was implemented when the number of SNPs available was larger than one. Wald ratio estimates of each individual SNP were combined in the IVW meta‐analysis, adjusting for heterogeneity.^25^ MR‐Egger and weighted median estimate (WME) was utilized, if there were three or more available SNPs. MR‐Egger appraises the association between exposure and outcome adjusted for any directional pleiotropy.^26^ In WME, the estimate will remain consistent even when up to 50% of the weight in the analysis comes from invalid SNPs, while in IVW, all of the SNPs are required to be valid IV.^27^ We also estimated the causal effect between the exposure and LUAD or LUSC (i.e., subgroup analysis), using wald ratio, IVW, MR‐Egger, and WME. In regard to multiple testing, Bonferroni correction was employed.^28^

Results of the evaluation of causal association were displayed as odds ratio (OR) between the exposure and outcome, as well as its 95% confidence interval (CI) and *p*‐value. Association was considered significant, when *p*‐value was less than 0.0008 (i.e., the Bonferroni corrected *p*‐value threshold, 0.05 / 60 putative traits), and considered suggestive, when *p*‐value was larger than 0.05 / 60 but less than 0.05.

### 2.6 Sensitivity analysis

Sensitivity analysis was performed to examine if there was any violation of the assumptions of MR or any other potential biases. Specifically, single SNP analysis, leave‐one‐out analysis, MR Egger, funnel plot, WME, and MR‐PRESSO were utilized. Single SNP analysis and leave‐one‐out analysis were conducted to find whether the estimate was driven by single SNP solely. MR‐Egger was used to assess whether there was any directional pleiotropy, to confirm that the genetic IV only affected the outcome through the exposure.^26^ MR estimates adjusted with directional pleiotropy were also provided by MR‐Egger. Dots will be symmetrically distributed in the funnel plot if there is no directional pleiotropy. WME is an approach for MR estimation in which even when up to 50% of the genetic IV utilized are invalid, the MR estimate will stay consistent.^27^ MR estimates from wald ratio, IVW, MR Egger, and WME were compared with each other to decide the robustness of the result. MR‐PRESSO was performed to identify the possible horizontal pleiotropy in MR analysis by its MR‐PRESSO global test.^29^ If there was pleiotropy, the MR‐PRESSO outlier test would be performed to figure out the potential outliers among the genetic IV, and to calculate the MR estimate which was corrected via outlier removal and thus was free of the detected pleiotropy. Finally, MR‐PRESSO distortion test would be conducted to assess whether there was significant difference between the MR estimates before and after the correction by removing the outliers.

Proportion of variance explained by the genetic IV (R^2^) and sample size were used to calculate the F‐statistic and power.^19^ The F‐statistic represents the strength of association between the genetic IVs and the exposure. If the F‐statistic is small, the genetic IVs utilized will be considered as weak IV. In other words, weak instrument bias may exist in this MR analysis. Meanwhile, power will also be small, suggesting that a relatively small‐to‐moderate causal relationship will not be detected (i.e., leading to false negative result), because the proportion of variance explained by the genetic IV utilized was not enough. Cut‐off point of F‐statistic and power were set to be 10 and 80% for the judgment of the strength of the genetic IVs.^20^, ^30^

### 2.7 Identification of potential intermediate factors

Some exposures were found to be significant risk factors of lung cancer after the Bonferroni correction. Some of them were in the same category. It was possible that they can be the intermediate factors in the causal relationship between other exposures and lung cancer. For this consideration, we performed a bidirectional MR analysis between the significant risk factors of lung cancer, utilizing wald ratio, IVW, MR‐Egger, and WME. In terms of sensitivity analysis, single SNP analysis, leave‐one‐out analysis, MR‐Egger, WME, and MR‐PRESSO were performed.

All of the data analysis in this study was performed using the package TwoSampleMR (version 0.4.25) in R (version 3.6.1).

## 3 RESULTS

We retained 46 putative modifiable risk factors of lung cancer, which were classified into 6 categories, 5 in factors of socioeconomic status (SES), 4 in factors of lifestyle, 29 in factors of dietary, 6 in cardiometabolic factors, 1 in developmental and growth factor, and 1 in inflammatory factor.

**FIGURE 2:**
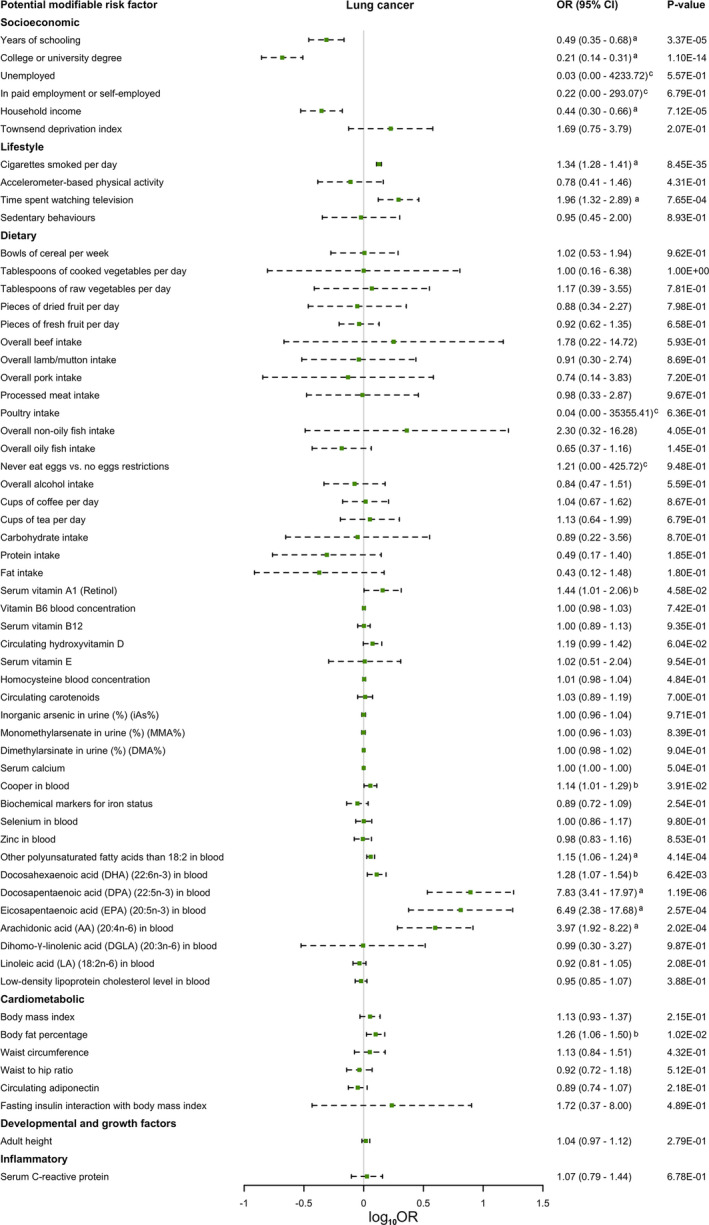
MR estimates [presented as log10(odds ratio)] of the relationship between the putative modifiable risk factors and lung cancer. (A) Significant risk factors of lung cancer; (B) Suggestive risk factors of lung cancer; (C) The line of the forest plot for this variable was not shown because its odds ratio was too large

**FIGURE 3:**
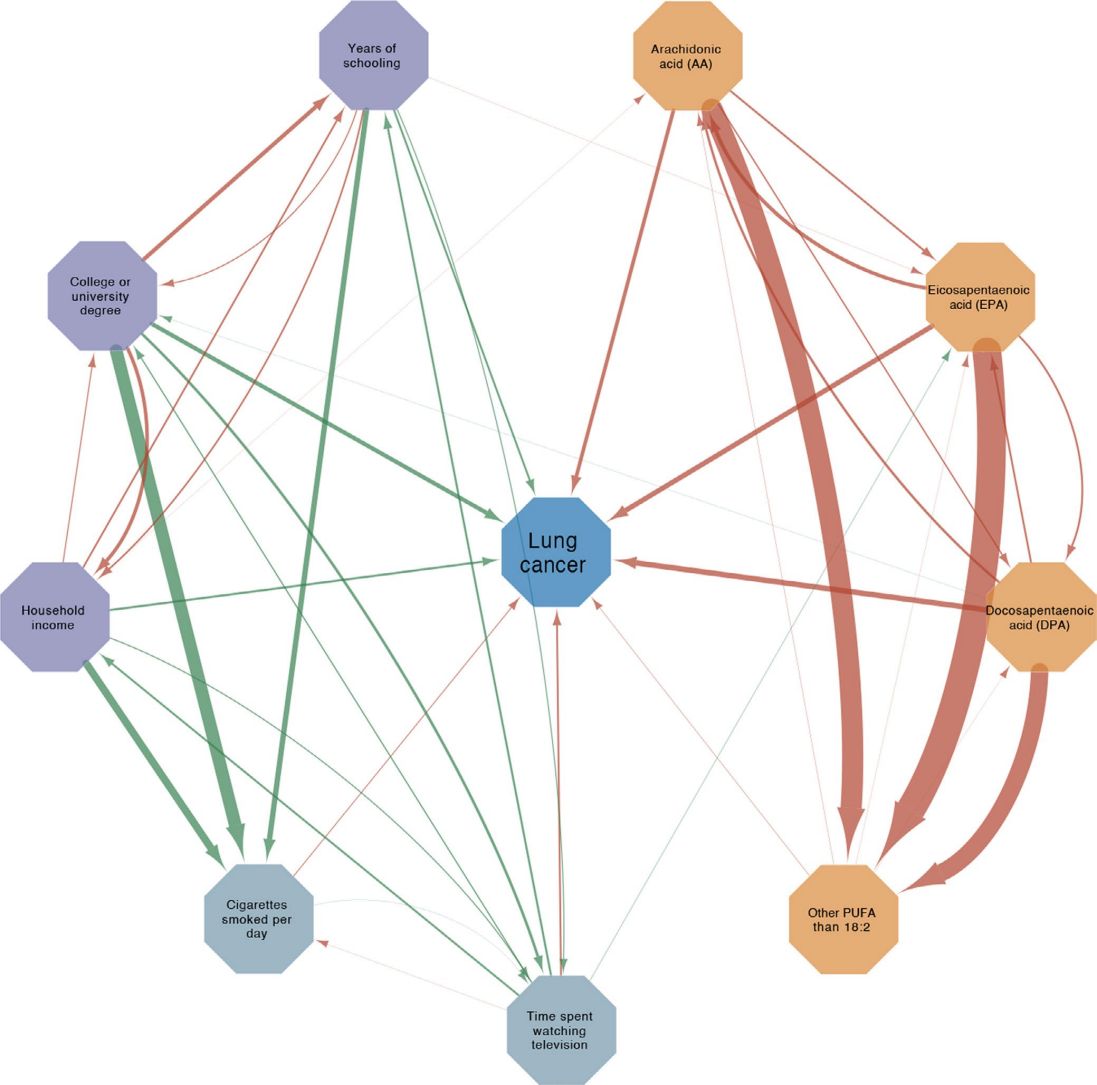
The relationship among all the significant risk factors and the relationship from the significant risk factors to lung cancer. i) The two variables connected by a line with arrow were correlated. If there was no line connecting them, the two variables were independent of each other. ii) Arrows of the lines indicated the direction of the relationship. For example, a line from years of schooling with an arrow toward time spent watching television represented how years of schooling would influence time spent watching television. iii) The thickness of the line represented the magnitude of the correlation strength. The thicker the line, the larger the magnitude was. iv) Variables connected with green line were inversely correlated. Variables connected with orange line were positively correlated

MR analysis between the 46 putative modifiable risk factors (60 traits included) and lung cancer, LUAD, and LUSC were conducted. MR estimates were presented in Figure 2, Table S4, Figure S4 and S5. In particular, the MR estimates between the significant risk factors of lung cancer were in Figure 3 and Table S9.

### 3.1 Socioeconomic status

MR provided significant evidence for the protective effect of years of schooling [OR (95% CI), 0.49 (0.35–0.68); *p*‐value <0.001], college or university degree [OR (95% CI), 0.21 (0.14–0.31); *p*‐value <0.001], and household income [OR (95% CI), 0.44 (0.30–0.66); *p*‐value <0.001] against lung cancer (Figure 2; Table S4). The association between college or university degree and lung cancer was consistent among IVW and WME. While the evidence for the MR estimates turned to be suggestive in WME for years of schooling and in MR‐Egger and WME for household income (Table S4; Figure S2). Driving SNPs was not found in the single SNP analysis and leave‐one‐out analysis (Table S5 and S6). MR‐Egger did not detect any directional pleiotropy (Table S7). Dots distributed symmetrically in the funnel plots and indicated no directional pleiotropy (Figure S3). Horizontal pleiotropy were found in MR‐PRESSO for years of schooling (<0.001), college or university degree (0.001) and household income (0.009) (Table S8). Outlying SNPs were identified for college or university degree (rs329122) and household income (rs2515919), while distortion test found no difference between the original and the corrected MR estimate (*p*‐value, 0.855 for college or university degree; *p*‐value, 0.701 for household income).

We noticed that SNPs available for unemployed (n = 2) and in paid employment or self‐employed (n = 1) were limited. Thus, Proportion of variance explained by the genetic IV (R^2^), power and F‐statistics for these two traits were relatively small (Table 2). In addition, R^2^ and power of Townsend deprivation index were also relatively small. Therefore, the null effect of unemployed, in paid employment or self‐employed, and Townsend deprivation index may have been affected by weak instrument bias. In other words, small‐to‐moderate causal effect between unemployed, in paid employment or self‐employed, and Townsend deprivation index and lung cancer may exist but was not detected in this study.

### 3.2 Lifestyle factors

Cigarettes smoked per day [OR (95% CI), 1.34 (1.28–1.41); *p*‐value <0.001] and time spent watching television [OR (95% CI), 1.96 (1.32–2.89); *p*‐value <0.001] were identified as significant risk factors of lung cancer (Figure 2; Table S4). In WME, time spent watching television had a suggestive relationship with lung cancer (Table S4; Figure S2). MR‐Egger showed no directional pleiotropy for time spent watching television (Table S7). The funnel plot of time spent watching television was also symmetric, indicating no directional pleiotropy (Figure S3). Rs6493583 was identified as an outlying SNP in the MR analysis of time spent watching television and lung cancer. However, the distortion test showed no significant difference in the MR estimates after the removal of rs6493583 (Table S8).

It is worth noting that only 1 SNP was available for cigarettes smoked per day, and thus only wald ratio was performed. Single SNP analysis, leave‐one‐out analysis, MR‐Egger, funnel plot, and MR‐PRESSO were not conducted in terms of cigarettes smoked per day. Power of cigarettes smoked per day did not exceed 80%, while F‐statistics and power for time spent watching television were sufficient (Table 2). We did not rule out the undetected small‐to‐moderate causal relationship between physical activity and time spent sedentary and lung cancer, regarding the insufficient power of them (Table 2).

### 3.3 Dietary factors

There was a significant causal relationship between other polyunsaturated fatty acids (PUFA) than 18:2 in blood [OR (95% CI), 1.15 (1.06–1.24); *p*‐value <0.001], docosapentaenoic acid (DPA) (22:5n‐3) in blood [OR (95% CI), 7.83 (3.41–17.97); *p*‐value <0.001], eicosapentaenoic acid (EPA) (20:5n‐3) in blood [OR (95% CI), 6.49 (2.38–17.68); *p*‐value <0.001], and arachidonic acid (AA) (20:4n‐6) in blood [OR (95% CI), 3.97 (1.92–8.22); *p*‐value <0.001] and lung cancer (Figure 2; Table S4). We also noted that serum vitamin A_1_ [OR (95% CI), 1.44 (1.01–2.06); *p*‐value, 0.046], copper in blood [OR (95% CI), 1.14 (1.01–1.29); *p*‐value, 0.04], and docosahexaenoic acid (DHA) (22:6n‐3) in blood [OR (95% CI), 1.28 (1.07–1.54); *p*‐value, 0.01] were suggestive risk factors of lung cancer. Other PUFA than 18:2 in blood was still a significant risk factor of lung cancer in WME. While in MR‐Egger, the association turned to be suggestive (Table S4; Figure S2). MR‐Egger detected no directional pleiotropy in the analysis between dietary and lung cancer (Table S7). However, the distribution of dots in the funnel plots of serum vitamin A_1_, copper, other PUFA than 18:2, and DHA in blood was asymmetric (Figure S3). The global test of MR‐PRESSO showed no horizontal pleiotropy in the analysis of all the significant and suggestive risk factors (Table S8). Although the horizontal pleiotropy was detected for some of the other unrelated dietary traits (i.e., vegetables, coffee, protein intake, serum calcium, linoleic acid (LA) (18:2n‐6) in blood, and low‐density lipoprotein cholesterol level in blood), there was no significant difference between the original and corrected MR estimate according to the distortion test.

Although the number of SNPs utilized for serum vitamin A_1_, copper, DPA, and EPA in blood was limited, the power and F‐statistics for all the significant and suggestive risk factors met the criteria, i.e., larger than 80% and 10, respectively (Table 2). However, R^2^ of 20 dietary traits was not available from the original article, thus leaving the power and F‐statistics of these traits unestimated. In other words, the existence of weak biases was possible.

### 3.4 Cardiometabolic, developmental and growth, and inflammatory factors

Body fat percentage was identified as a suggestive risk factor of lung cancer [OR (95% CI), 1.26 (1.06–1.50); *p*‐value, 0.01] (Figure 2; Table S4). Directional pleiotropy was not detected in all the traits in MR‐Egger analysis (Table S7). While funnel plots of waist circumference, circulating adiponectin, fasting insulin interaction with body mass index (BMI), and serum C‐reactive protein did not seem symmetric (Figure S3). Horizontal pleiotropy and outlying SNP were reported by MR‐PRESSO for body fat percentage, but the distortion test showed no significant difference after removing the outlying SNP (Table S8). The distortion test was also insignificant for other traits except for adult height (*p*‐value of distortion test, 0.03). However, the corrected result of adult height still indicated no causal relationship with lung cancer (*p*‐value, 0.96). Power and F‐statistics of all the traits were sufficient, despite the fact that the number of SNPs available of serum C‐reactive protein was only 3 (Table 2).

### 3.5 Subgroup analysis for LUAD and LUSC

We also analyzed the causal relationship between modifiable risk factors and LUAD and LUSC. The significant relationships with LUAD were discovered in college or university degree [OR (95% CI), 0.26 (0.15–0.44); *p*‐value, <0.001], cigarettes smoked per day [OR (95% CI), 1.32 (1.23–1.42); *p*‐value, <0.001], DPA [OR (95% CI), 15.22 (4.33–53.58); *p*‐value, <0.001], and AA [OR (95% CI), 6.66 (2.25–19.74); *p*‐value, <0.001] (Table S4; Figure S4). Suggestive risk factors of LUAD were household income [OR (95% CI), 0.03 (0.00–0.25); *p*‐value, 0.003], time spent watching television [OR (95% CI), 1.81 (1.02–3.21); *p*‐value, 0.04], fat intake [OR (95% CI), 0.20 (0.06–0.72); *p*‐value, 0.01], serum vitamin B_12_ [OR (95% CI), 1.24 (1.04–1.49); *p*‐value, 0.02], other PUFA than 18:2 [OR (95% CI), 1.20 (1.07–1.35); *p*‐value, 0.002], DHA [OR (95% CI), 1.38 (1.03–1.85); *p*‐value, 0.03], and EPA [OR (95% CI), 12.94 (2.88–58.14); *p*‐value, <0.001].

Years of schooling [OR (95% CI), 0.40 (0.26–0.62); *p*‐value, <0.001], college or university degree [OR (95% CI), 0.14 (0.08–0.25); *p*‐value, <0.001], household income [OR (95% CI), 0.41 (0.24–0.68); *p*‐value, <0.001], cigarettes smoked per day [OR (95% CI), 1.34 (1.25–1.44); *p*‐value, <0.001], time spent watching television [OR (95% CI), 3.03 (1.71–5.35); *p*‐value, <0.001], and DPA [OR (95% CI), 8.97 (2.59–31.01); *p*‐value, <0.001] remained significant risk factors of LUSC after the Bonferroni correction (Table S4; Figure S5). Biochemical markers for iron status [OR (95% CI), 0.75 (0.61–0.91); *p*‐value, 0.005], other PUFA than 18:2 [OR (95% CI), 1.16 (1.03–1.30); *p*‐value, 0.01], EPA [OR (95% CI), 11.33 (2.52–50.97); *p*‐value, 0.002], AA [OR (95% CI), 5.41 (1.81–16.18); *p*‐value, 0.003], BMI [OR (95% CI), 1.37 (1.02–1.85); *p*‐value, 0.04], and body fat percentage [OR (95% CI), 1.32 (1.02–1.72); *p*‐value, 0.03] were identified as suggestive risk factors of LUSC.

MR estimates of LUAD and LUSC by other approaches were displayed in Table S4. MR‐Egger detected directional pleiotropy between household income (intercept, 0.06; *p*‐value, 0.01) and circulating adiponectin (intercept, 0.04; *p*‐value, 0.01) and LUAD (Table S7). Thus, the MR estimates of household income reported above and that of circulating adiponectin in Figure S4 were obtained by MR‐Egger to adjust for the observed directional pleiotropy. MR‐PRESSO found horizontal pleiotropy in the MR between time spent watching television and LUAD, college or university degree, BMI, and body fat percentage and LUSC (Table S8). However, the distortion test of MR‐PRESSO showed no significant difference between the original MR estimates and the corrected ones for all the tested traits of LUAD and LUSC.

### 3.6 Bidirectional MR among the significant modifiable risk factors of lung cancer

We performed bidirectional MR analyses between significant modifiable risk factors of lung cancer to figure out whether there were intermediate factors in the significant relationship identified. All the significant traits were included, three traits of SES (years of schooling, college or university degree, and household income), two traits of lifestyle (cigarettes smoked per day and time spent watching television), and four traits related to PUFA (other PUFA than 18:2, DPA, EPA, and AA in blood).

We found that all the three significant traits of SES were positively correlated with each other in both directions (Figure 3, Table S9). Years of schooling, college or university degree, and household income were all indicators of both less cigarettes smoked per day as well as less time spent watching television. Inversely, time spent watching television was conversely associated with years of schooling, college or university degree, and household income, but positively associated with cigarettes smoked per day. Similarly, all the four significant traits related to PUFA were positively associated with each other in both directions. Directional pleiotropy was found in the MR analysis from time spent watching television to cigarettes smoked per day by MR‐Egger. (intercept, −0.12; *p*‐value, 0.04). Considering the directional pleiotropy detected, the claimed positive effect of time spent watching television on cigarettes smoked was based on the MR‐Egger estimate. According to the result of MR‐PRESSO, the *p*‐value of the distortion test between time spent watching television and AA in blood was 0.043. But both the original MR estimate (*p*‐value, 0.54) and the outlier‐corrected MR estimate (*p*‐value, 0.93) suggested no association between them.

## 4 DISCUSSION

In this study, we analyzed 46 putative modifiable risk factors of lung cancer utilizing MR analysis. We noted that years of schooling, college or university degree, and household income had significant protective effects on lung cancer. This study also provided significant evidence for the positive association of lung cancer with cigarettes smoked per day, time spent watching television, other PUFA than 18:2, DPA, EPA, and AA in blood. We also noted suggestive associations between raised serum vitamin A_1_, copper and, DHA in blood as well as body fat percentage and increased risk of lung cancer. The bidirectional MR among the significant traits above indicated that they may be intermediate factors of each other.

Our findings show that higher educational attainment and household income decreased lung cancer risk were concordant with the findings of previous conventional observational studies.^31^, ^32^, ^33^, ^34^, ^35^ In fact, we observed the phenomenon that higher educational attainment was causally associated with a lower risk of lung cancer by using the framework of two‐sample MR previously, while household income was reported in this study for the first time (Table S10).^17^ SES inequalities in lung cancer incidence have long been noted. Data from the SYNERGY study and the Canadian Census Cohort have shown that SES remains a risk factor for lung cancer.^33^ We further investigated the intermediary mechanisms between SES factors, underlying their observed relationship with lung cancer. The positive causal relationship among significant SES factors (years of schooling, college degree, and household income) indicated that they could influence each other and lower the risk of lung cancer as a whole. Moreover, the significant SES factors also affected the lifestyle factors (i.e., smoking and watching television), which were significant risk factors of lung cancer.^17^, ^36^, ^37^, ^38^

Among the lifestyle factors analyzed in this study, cigarettes smoked per day and time spent watching television were identified as significant risk factors of lung cancer. The effect of smoking on lung cancer has been well established.^6^, ^39^, ^40^ And cigarette cessation has resulted in a decline in lung cancer incidence.^41^ In this study, we testified the causal effect of smoking on lung cancer, which was supported by Larsson et al.^42^ Physical activity has been classified as a protective factor of lung cancer by WCRF and AICR, with limited suggestive evidence.^6^ An inverse association between physical activity and lung cancer was also found in a meta‐analysis.^43^ However, the MR estimate in this study and that from a previous MR analysis indicated that lung cancer was independent of physical activity.^44^ The protective effect of physical activity observed in observational studies may have been influenced by confounding effect and information bias. In this MR study, we verified the raised lung cancer incidence with increased time spent watching television, which was observed by Schmid et al. in a meta‐analysis.^45^ This study was the first MR analysis concerning time spent watching television and lung cancer. Reducing time spent watching television may be beneficial in preventing lung cancer. Moreover, according to our results, the increased time spent watching television declined the years of schooling and household income, lowered the probability of getting a college or university degree, and increased the number of cigarettes smoked per day. Despite the direct causal relationship between time spent watching television and lung cancer, we did not rule out the possibility that time spent watching television also increased lung cancer risk by lowering the SES and promoting smoking.

Diet and nutrition factors have been attached with great importance. According to the WCRF/AICR report, there is limited evidence to support the role of diet and nutrition in the development of lung cancer, except for arsenic in drinking water and high‐dose beta‐carotene supplements which have convincing evidence.^6^ We utilized two‐sample MR to systematically assess the causality between dietary and lung cancer for the first time. Most of the factors were found to be unrelated to lung cancer. Although we did not observe the causal relationship between arsenic related metabolites in urine and lung cancer through this MR, we could not deny the convincing fact that arsenic is a well‐known carcinogen for lung cancer, because arsenic metabolism cannot fully proxy arsenic exposure.^46^, ^47^, ^48^ Our results for vegetables and fruits were contrary to previous evidence.^49^ Vieira et al. showed an 8%‐18% decreased risk of lung cancer with higher consumption of fruits and vegetables, but the relationship may be confounded by smoking status.^49^ In our study, intake of meat did not impact lung cancer risk regardless of the type of meat consumed, which is also in contrast to previous research.^50^

The anticancer effect of vitamin supplements is another issue worthy of attention. Our results in combination with previous research did not provide evidence that circulating vitamins had a protective role in lung cancer.^51^, ^52^, ^53^ It should be cautious to recommend vitamin supplementation as a preventive strategy for lung cancer. Moreover, serum vitamins A_1_ and vitamin B_12_ even had suggestive tendencies to promote the development of lung cancer.^54^ Our previous MR study of blood trace minerals had indicated that genetically predicted higher blood copper level was causally associated with a greater risk of lung cancer, which is consistent with the previous study.^14^, ^55^ The potential mechanism may involve the oncogenic BRAF signal pathway.^56^ Furthermore, higher copper level increases oxidative stress, damages large biomolecules, and ultimately leads to oncogenesis.^57^ The MR study performed by Liu et al. reported DPA, a kind of PUFA, was linked to the risk of lung cancer.^13^ We extended the MR analysis to multiple types of PUFA, and found inconsistent results with previous studies.^58^, ^59^, ^60^ The potential adverse effects of other PUFA than 18:2, DHA, DPA, EPA, and AA on lung cancer patients should be considered when developing dietary guidelines on cancer prevention.

The MR analysis suggested a distinct causal effect of BMI and body fat percentage on LUSC and LUAD, with evidence of an increased risk of LUSC and a null relationship with LUAD. This finding is consistent with previous MR studies, and highlighted the histologic‐specific impact of BMI.^61^, ^62^, ^63^ In view of this, we have also compared the MR results of LUAD and those from LUSC. Other modifiable risk factors have consistent risk trends within groups, except for BMI and body fat percentage. Previous MR studies from Transdisciplinary Research in Cancer of the Lung (TRICL) and East Asian populations indicated that increased height may have a causal role in lung cancer.^64^, ^65^ However, in our study, height had nothing to do with lung cancer. The possible reason we considered is that the population source of the GWAS data is different, and this also reminded us to pay attention to the influence of race in subsequent research.

Our study has several important strengths. We conducted the first two‐sample MR study to systematically draft the modifiable risk factors atlas of lung cancer by using data from large GWAS studies. All 46 risk factors included in this analysis were selected based on our systematic review of previous meta‐analyses and the WCRF/AICR report. The risk factors selected were potentially implicated in lung cancer development with varying degrees of evidence. Many factors have not previously been included in MR analyses of lung cancer, including those proven to be significant risk factors of lung cancer in this study (i.e., college or university degree, household income, time spent watching television, other PUFA than 18:2, EPA, and AA in blood). The use of MR framework can prevent the residual effect of confounders and reverse causality that are commonly present in conventional observational epidemiological studies. Moreover, we displayed the network among significant risk factors and lung cancer for the first time. We also found no significant difference in risk factors between LUAD and LUSC, except for BMI and body fat percentage.

However, there were still some limitations in this study. First, false negative results may exist in this study, because of the weak instrument bias of some traits, regarding the limited number of SNPs available and R2 and the insufficient power and F‐statistics.^20^ In addition, for some traits, especially those related to dietary, R^2^ was not available in the original article, leaving the weak instrument bias unestimated.^66^ Second, IVW, MR‐Egger, WME, leave‐one‐out analysis, and MR‐PRESSO were not performed, with the limitation due to the limited number of SNPs available for some traits. Third, GWAS data used for exposure and outcome in this study was the same as those used in previous MR studies, such as years of schooling and lung cancer.^17^ To some extent, this reduced the innovation of the research. However, for the first time, we analyzed the causal relationship between many other modifiable risk factors and lung cancer. We also assessed the observed association utilizing Bonferroni correction for this multivariable study. Fourth, robust genetic IVs were not accessible for many other modifiable risk factors and thus we did not include these factors in this study. GWAS concerning these factors were warranted, with which MR analysis between these potential risk factors and lung cancer would be possible. Last but not least, the generalizability of the conclusion was restricted by three issues.^67^ First, GWAS data used were mainly based on European population. External validity in other populations is necessary. Second, the utilization of genetic IVs represented that the exposure to the trait was possibly lifelong, which can be different from the actual situation. Similarly, the actual levels of exposure to the trait may also influence the application of our conclusion.

## 5 CONCLUSIONS

With the utilization of MR analysis, we provided the evidence for the relationship between previously reported risk factors and lung cancer from the aspect of causation. We identified several modifiable targets for primary prevention of lung cancer, concerning socioeconomic status, lifestyle, dietary, and obesity.

## CONFLICT OF INTEREST

All authors have no conflicts of interested to declare.

## AUTHORS’ CONTRIBUTIONS

Study concept and design: Jiayi Shen, Huaqiang Zhou, Jiaqing Liu, Yan Huang, Li Zhang. Acquisition of data: Jiayi Shen, Huaqiang Zhou, Jiaqing Liu. Analysis of data: Jiayi Shen, Huaqiang Zhou, Jiaqing Liu. Drafting of the manuscript: Huaqiang Zhou, Jiayi Shen, Jiaqing Liu with input of all authors. Critical revision of the manuscript for important intellectual content: All authors.

## ETHICS APPROVAL AND CONSENT TO PARTICIPATE

N/A, the need for approval was waived because the present MR analysis was based on anonymous summary data from previous studies.

## CONSENT FOR PUBLICATION

All authors of this paper have read and approved the final version submitted.

## Supporting information

Fig S1‐5

Table S1‐10

## Data Availability

The dataset(s) supporting the conclusions of this article is(are) available from corresponding GWAS consortium.
