# Supplementary material for: A modifiable risk factors atlas of lung cancer: A Mendelian randomization study
Source: Cancer Med. 2021 Jun 2;10(13):4587–603. doi: 10.1002/cam4.4015 (PMC8267159; doi:10.1002/cam4.4015)

Supplementary Figures

Supplementary Figure 1. Principles and assumptions of MR analysis.

Principle of MR: i) SNPs (single nucleotide polymorphisms) were utilized as genetic instrumental variables (IVs) of the exposure (putative modifiable risk factors of lung cancer in this study); ii) Effects of the genetic IVs on exposure and outcome were assessed separately; iii) Effects of the genetic IVs on exposure and outcome were compared to obtain the MR estimate.

Assumptions of MR: (A) The IVs is associated with the risk factor (Relevance); (B) The IVs affects the outcome only through the risk factor (Exclusion restriction); and (C) The IVs is not associated with any confounders (Independent)

Supplementary Figure 2. Scatter plots of the MR analysis between putative modifiable risk factors and lung cancer.

Supplementary Figure 3. Funnel plots for the assessment of directional pleiotropy in the MR analysis between putative modifiable risk factors and lung cancer.

Supplementary Figure 4. MR estimates [presented as log10(odds ratio)] of the relationship between the putative modifiable risk factors and lung adenocarcinoma.

a) Significant risk factors of lung cancer; b) Suggestive risk factors of lung cancer; c) The line of the forest plot for this variable was not shown because its odds ratio was too large. d) MR estimate of this variable was derived from MR Egger, for the adjustment of the detected directional pleiotropy.

Supplementary Figure 5. MR estimates [presented as log10(odds ratio)] of the relationship between the putative modifiable risk factors and lung squamous cell carcinoma.

a) Significant risk factors of lung cancer; b) Suggestive risk factors of lung cancer; c) The line of the forest plot for this variable was not shown because its odds ratio was too large.

Supplementary Figure 1. Principles and assumptions of MR analysis.

Principle of MR: i) SNPs (single nucleotide polymorphisms) were utilized as genetic instrumental variables (IVs) of the exposure (putative modifiable risk factors of lung cancer in this study); ii) Effects of the genetic IVs on exposure and outcome were assessed separately; iii) Effects of the genetic IVs on exposure and outcome were compared to obtain the MR estimate.

Assumptions of MR: (A) The IVs is associated with the risk factor (Relevance); (B) The IVs affects the outcome only through the risk factor (Exclusion restriction); and (C) The IVs is not associated with any confounders (Independent)

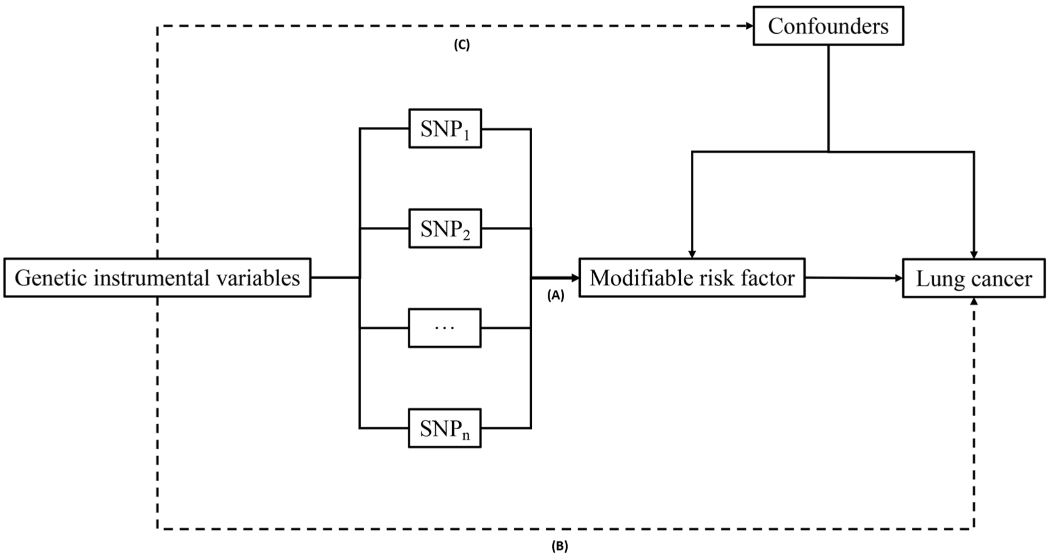

Supplementary figure 2. Scatter plots of the MR analysis between putative modifiable risk factors and lung cancer.

1/7

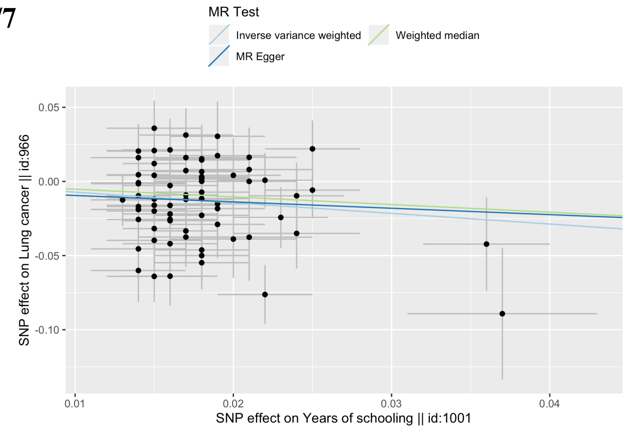

Years of schooling

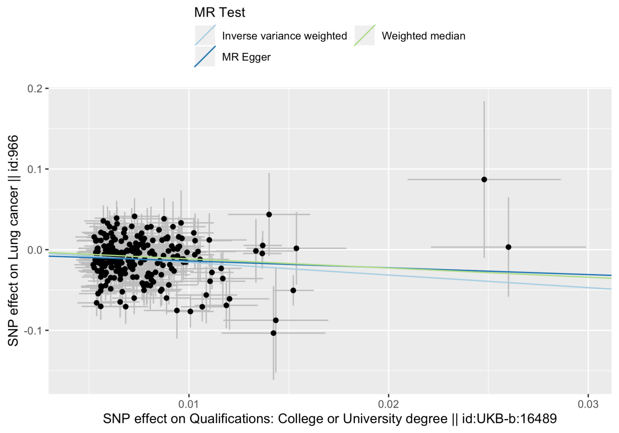

College or university degree

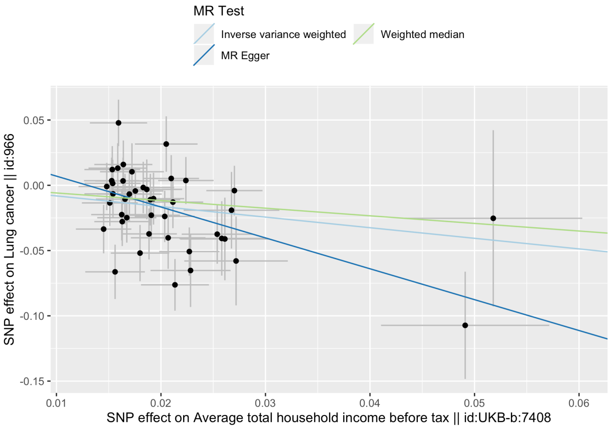

Household income

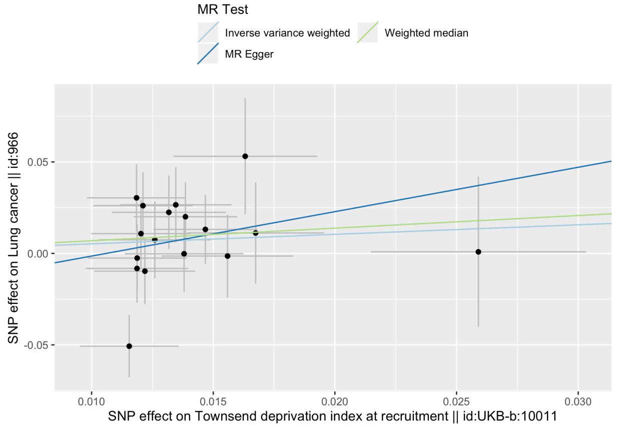

Townsend deprivation index

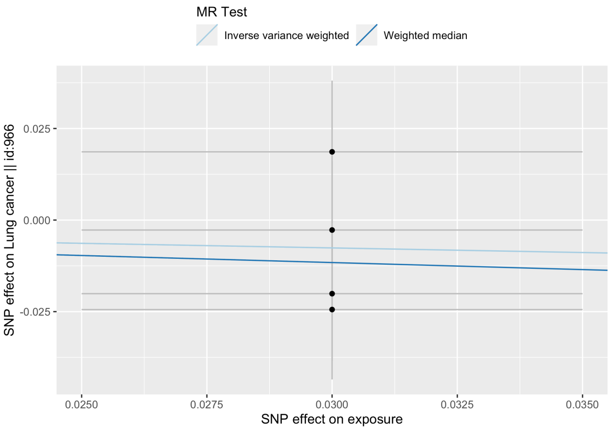

Accelerometer-based physical activity

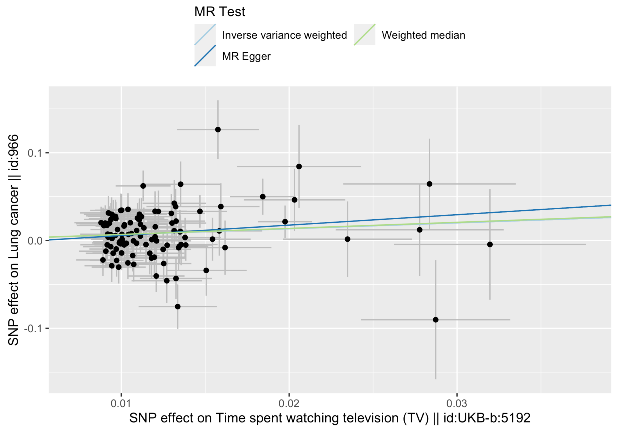

Time spent watching television

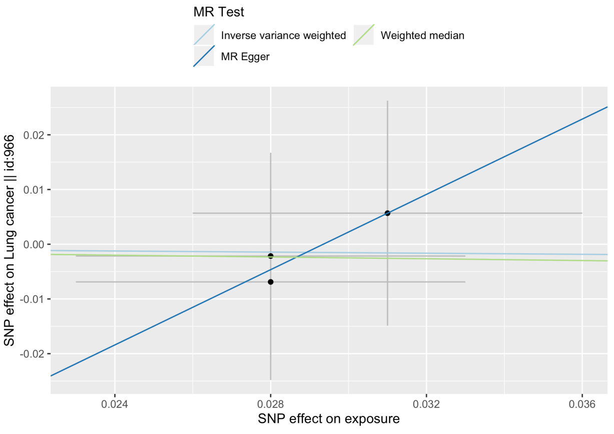

Sedentary behaviours

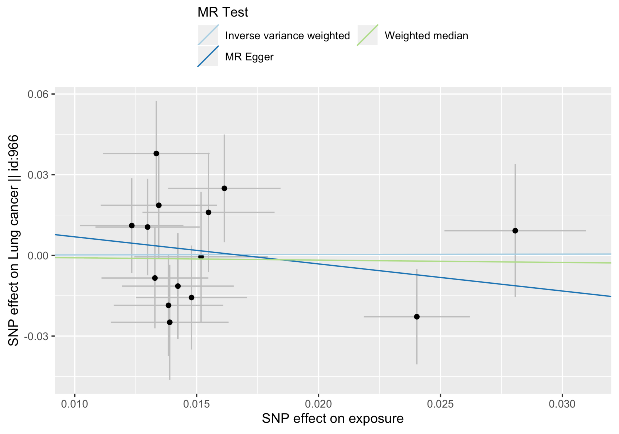

Bowls of cereal per week

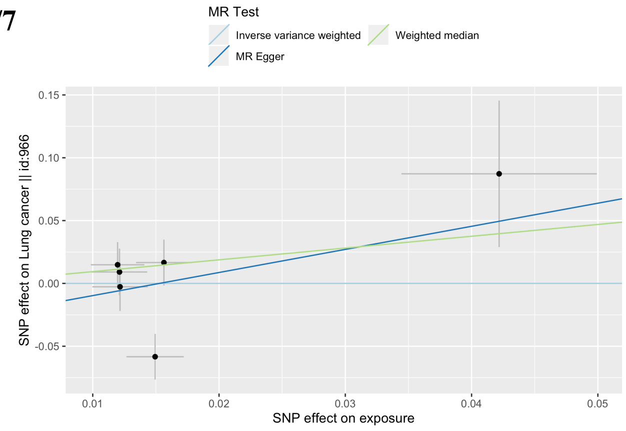

Tablespoons of cooked vegetables per day

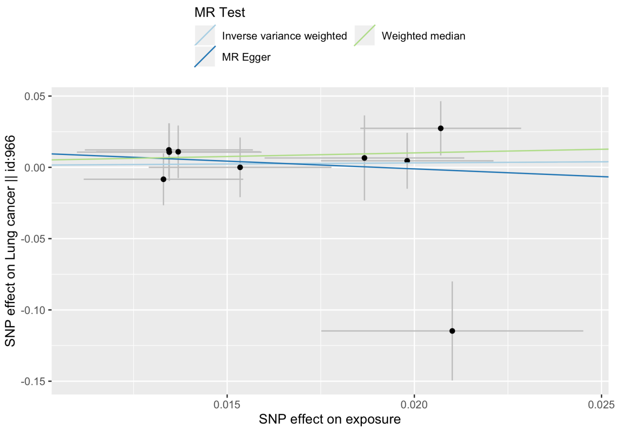

Tablespoons of raw vegetables per day

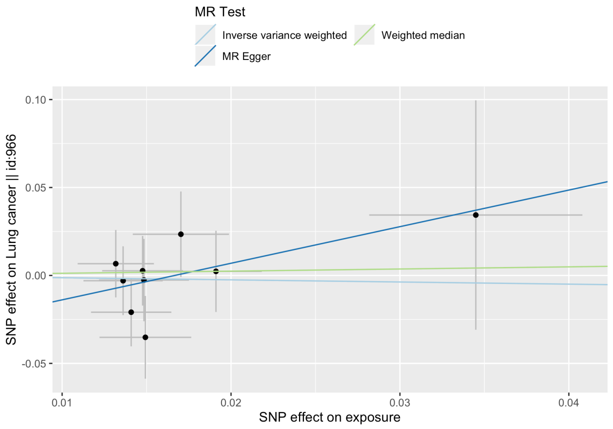

Pieces of dried fruit per day

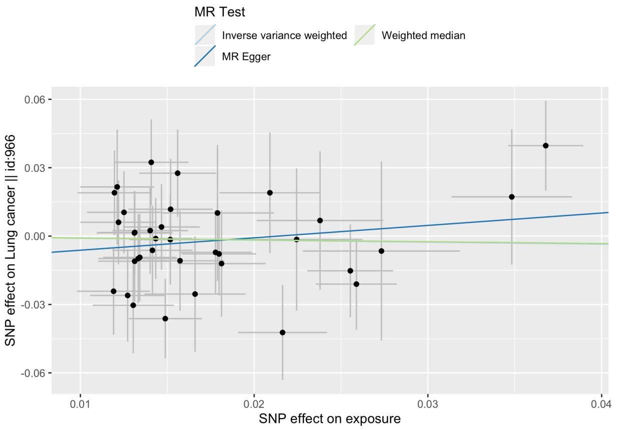

Pieces of fresh fruit per day

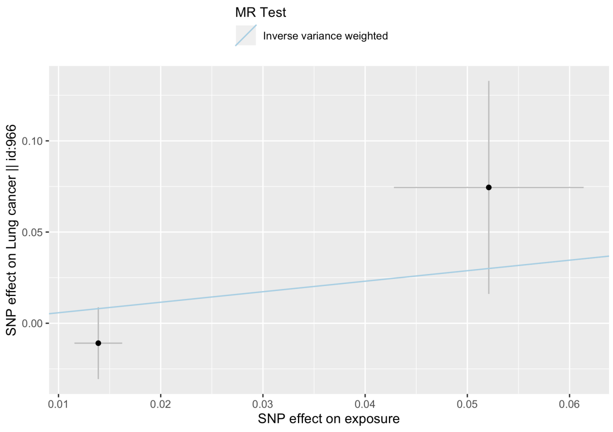

Overall beef intake

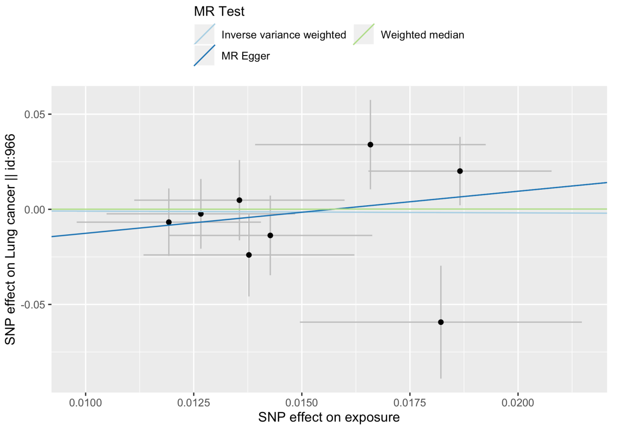

Overall lamb/mutton intake

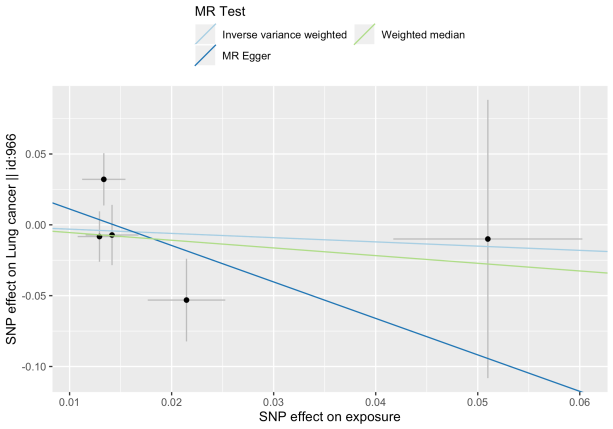

Overall pork intake

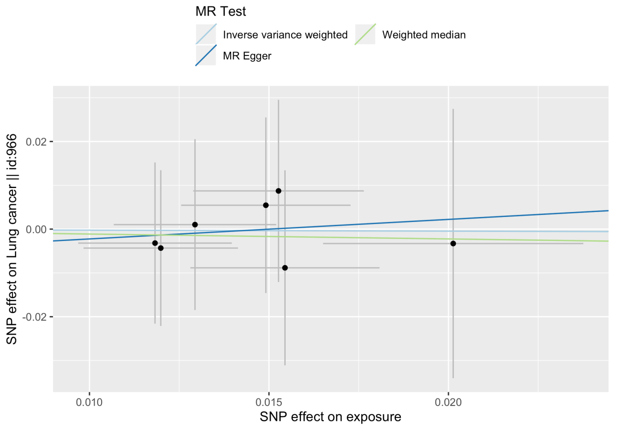

Processed meat intake

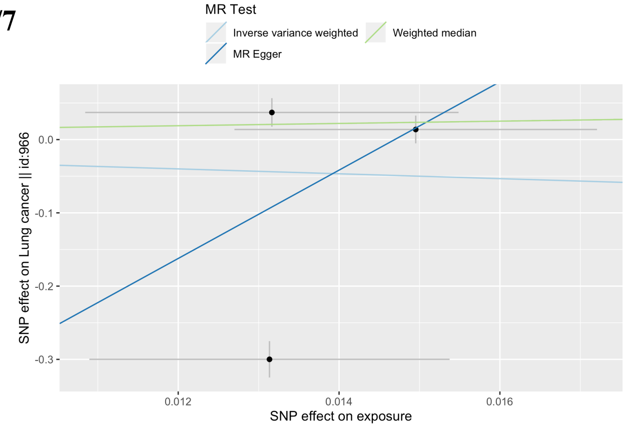

Poultry intake

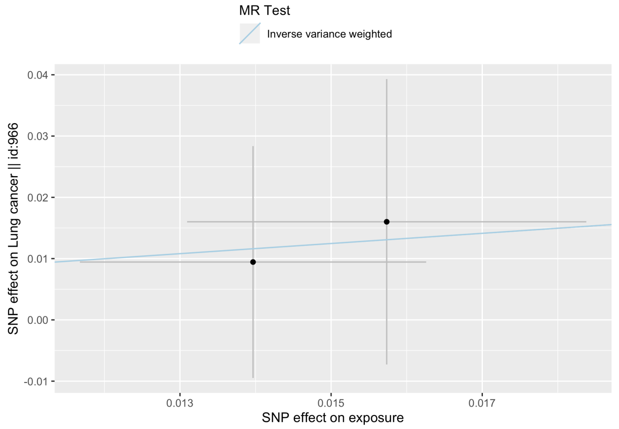

Overall non-oily fish intake

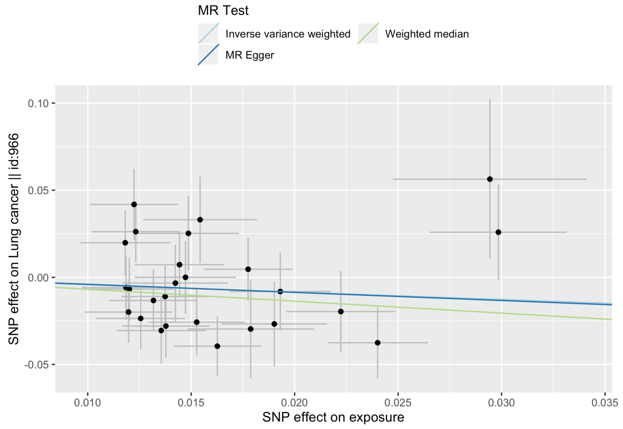

Overall oily fish intake

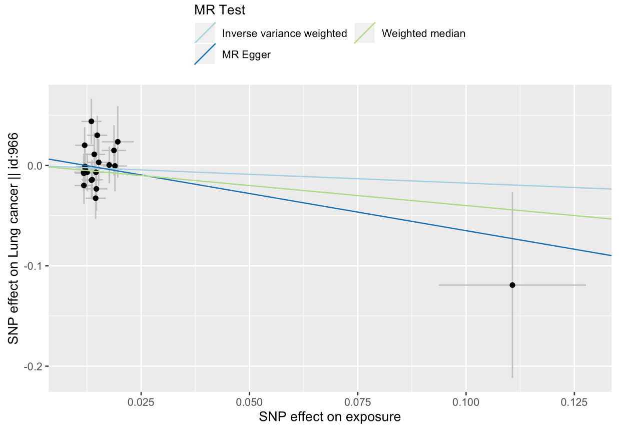

Overall alcohol intake

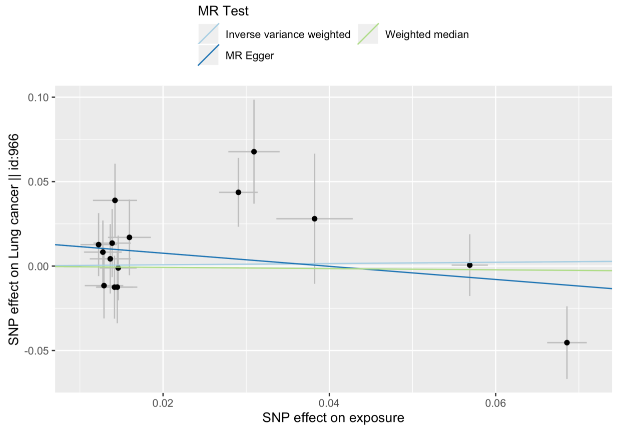

Cups of coffee per day

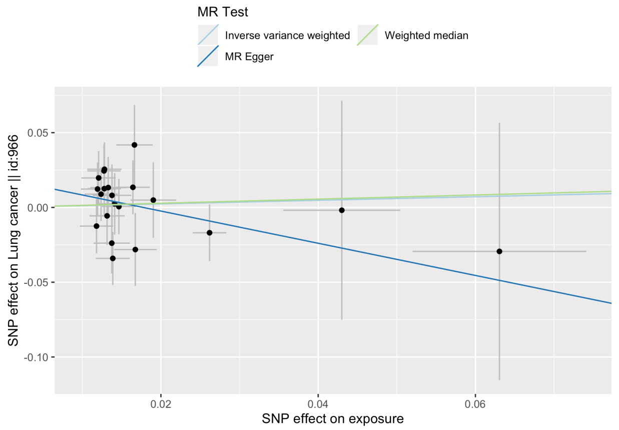

Cups of tea per day

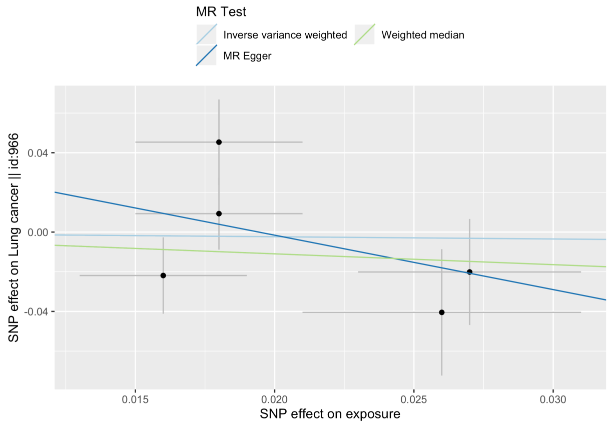

Carbohydrate intake

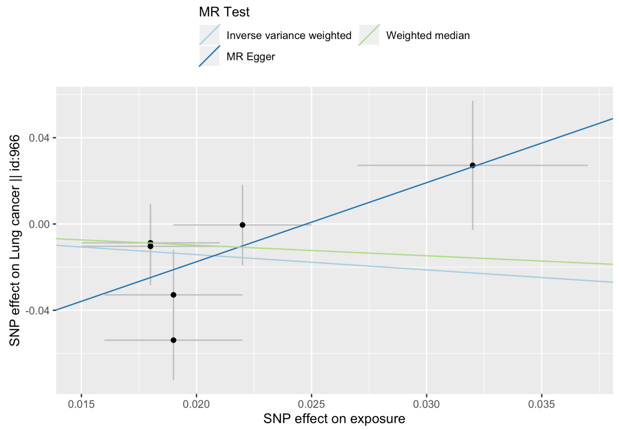

Protein intake

4/7

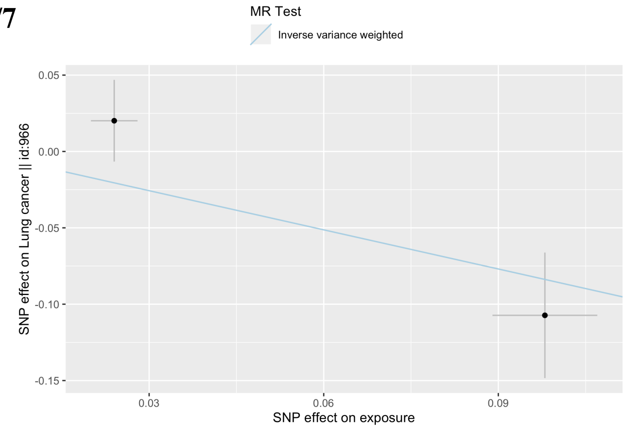

Fat intake

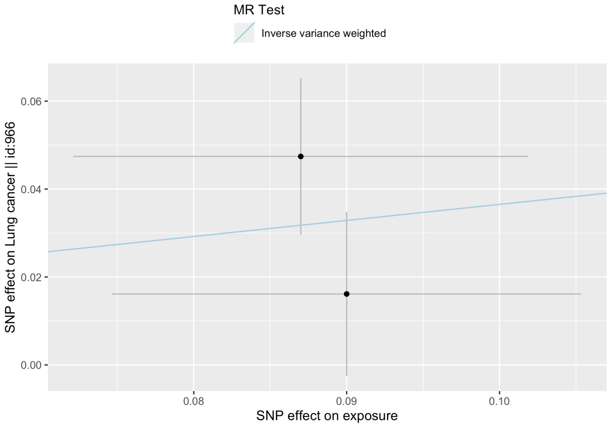

Serum vitamin A1 (Retinol)

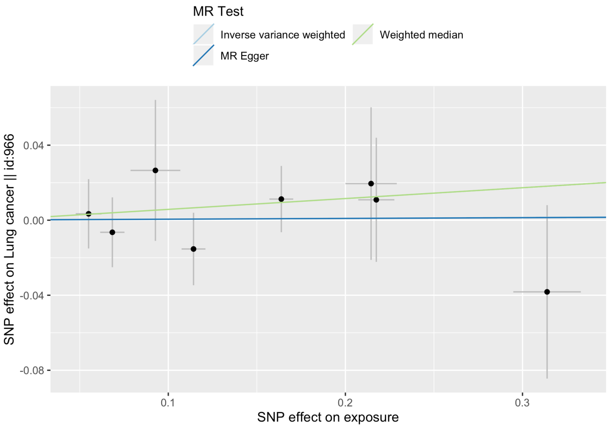

Serum vitamin B12

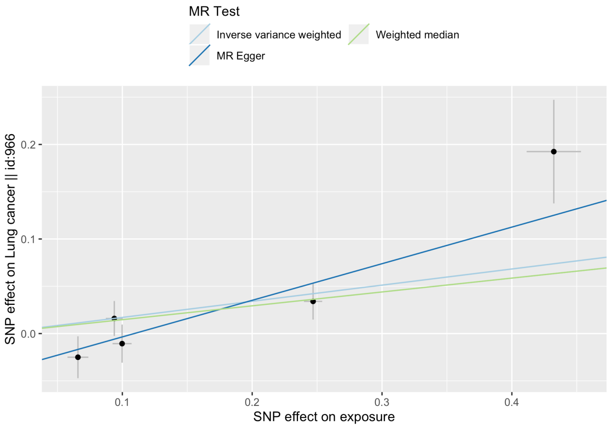

Circulating hydroxyvitamin D

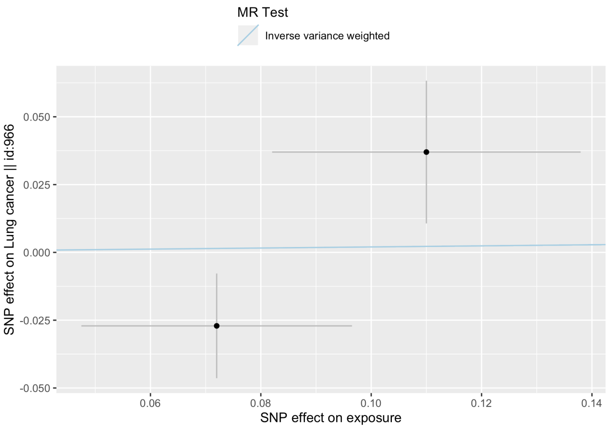

Serum vitamin E

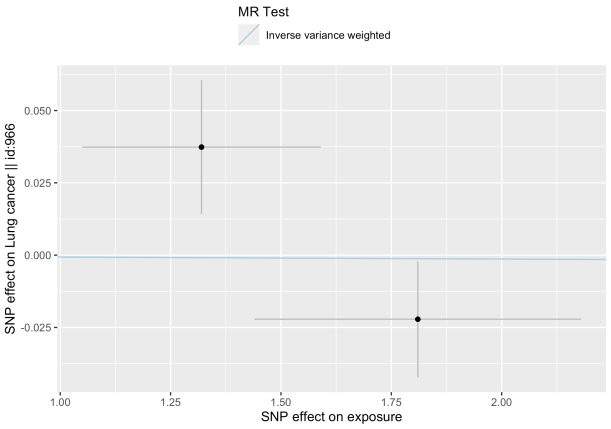

Inorganic arsenic in urine (%) (iAs%)

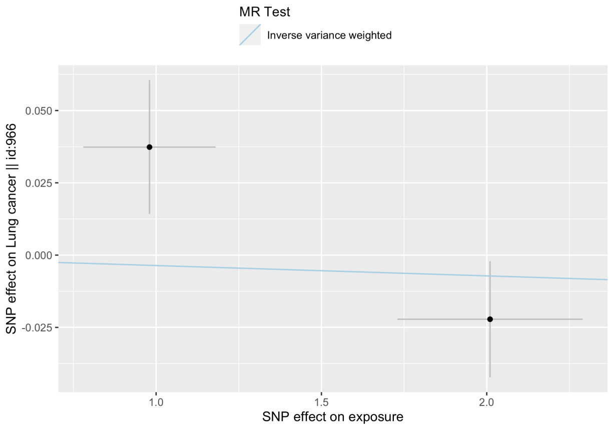

Monomethylarsenate in urine (%) (MMA%)

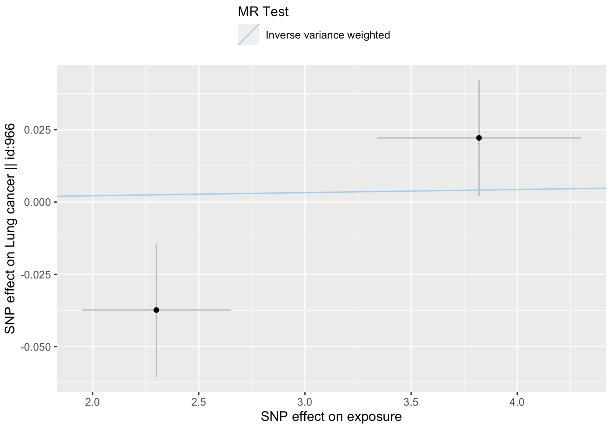

Dimethylarsinate in urine (%) (DMA%)

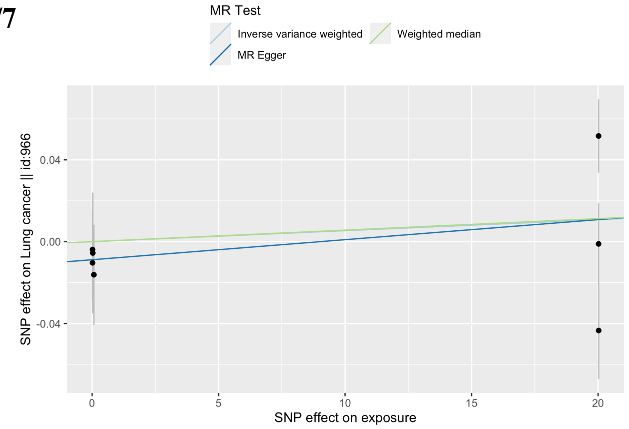

Serum calcium

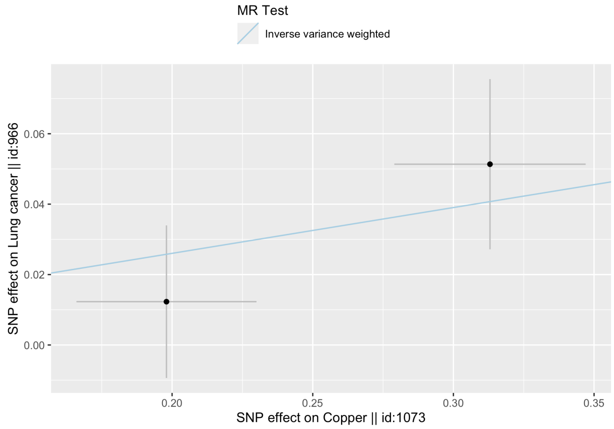

Cooper in blood

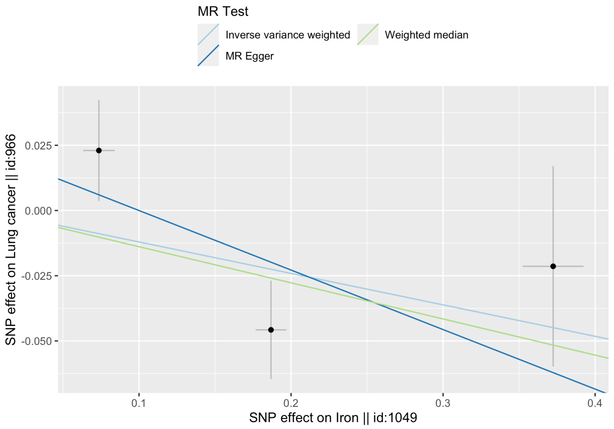

Biochemical markers for iron status

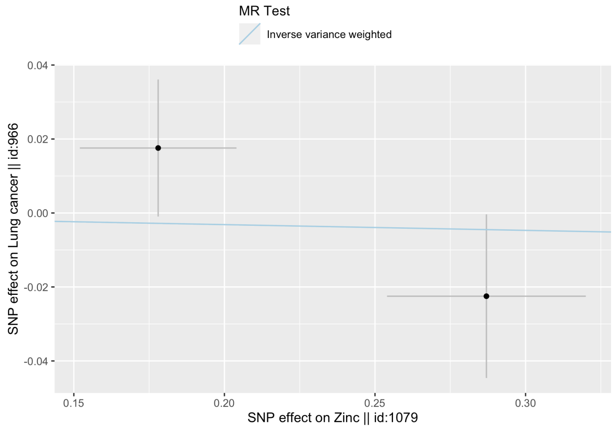

Zinc in blood

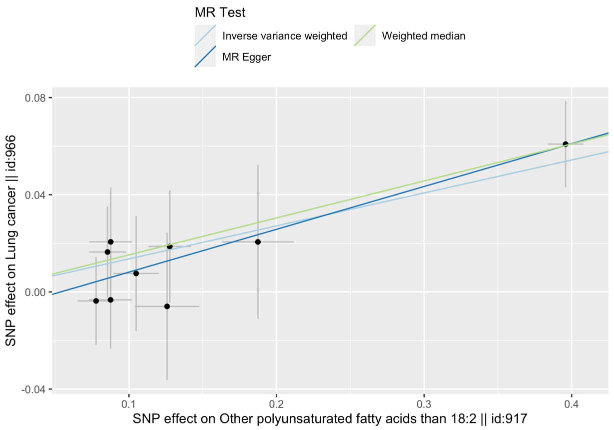

Other polyunsaturated fatty acids than 18:2 in blood

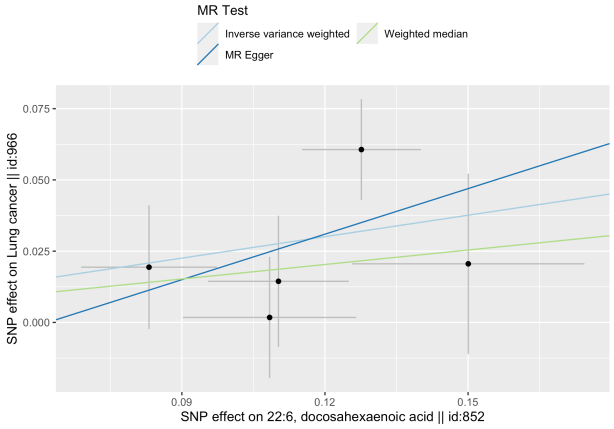

Docosahexaenoic acid (DHA) (22:6n-3) in blood

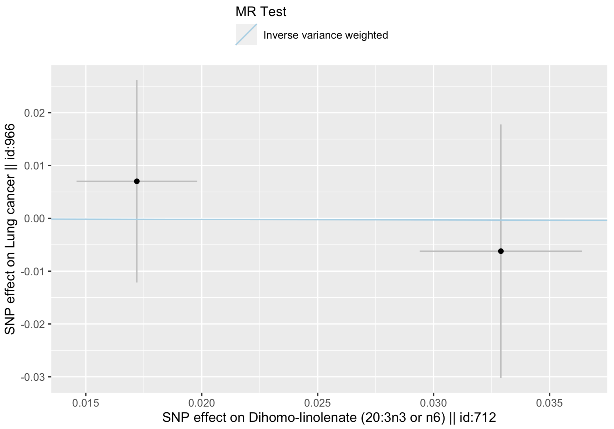

Dihomo-γ-linolenic acid (DGLA) (20:3n-6) in blood

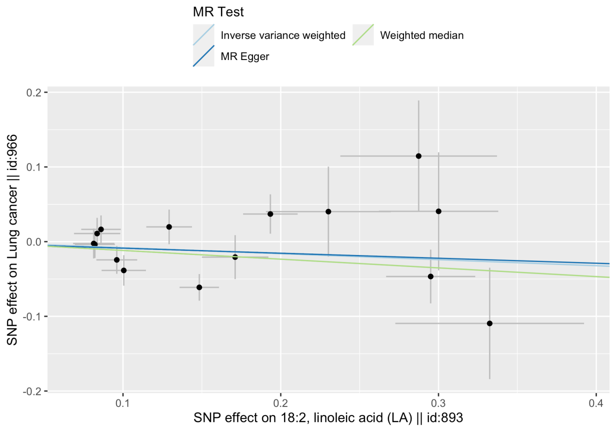

Linoleic acid (LA) (18:2n-6) in blood

6/7

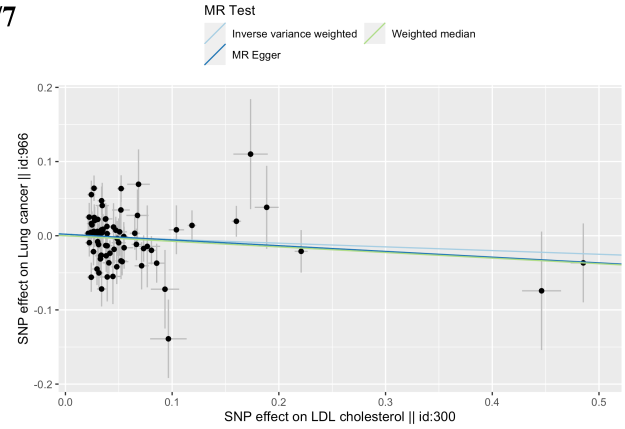

Low-density lipoprotein cholesterol level in blood

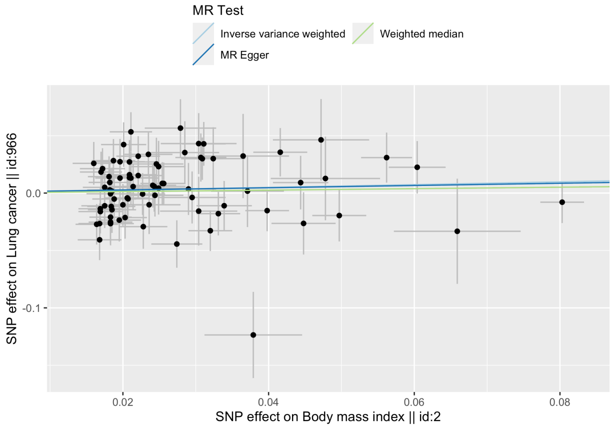

Body mass index

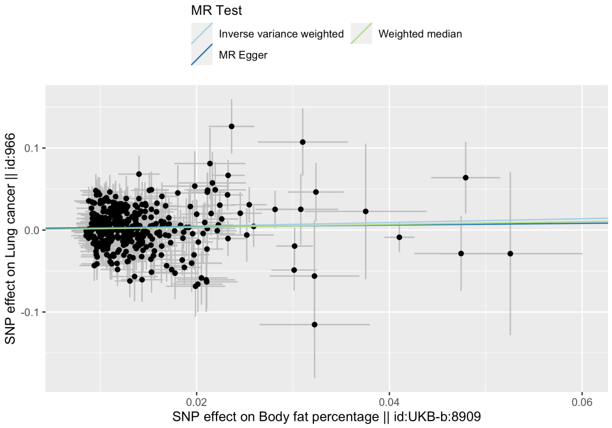

Body fat percentage

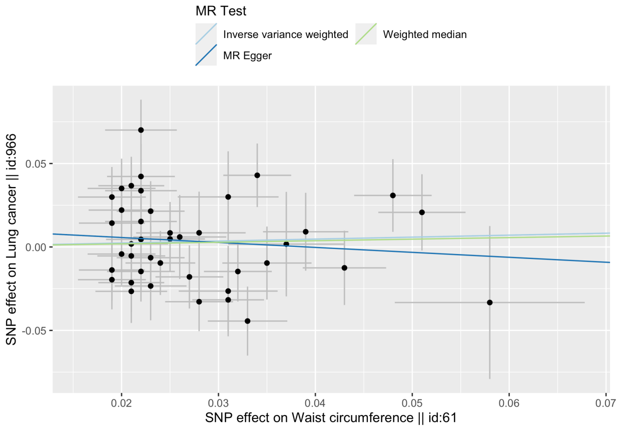

Waist circumference

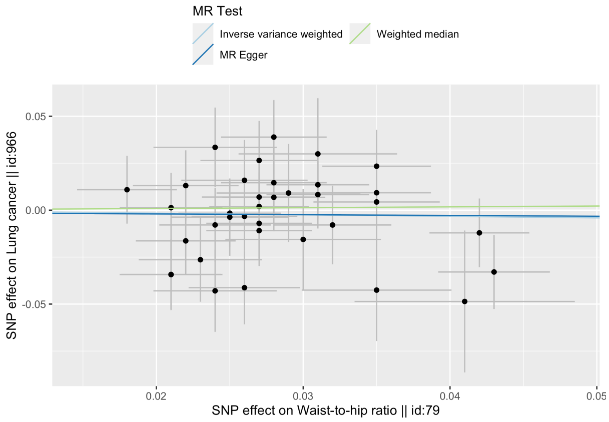

Waist to hip ratio

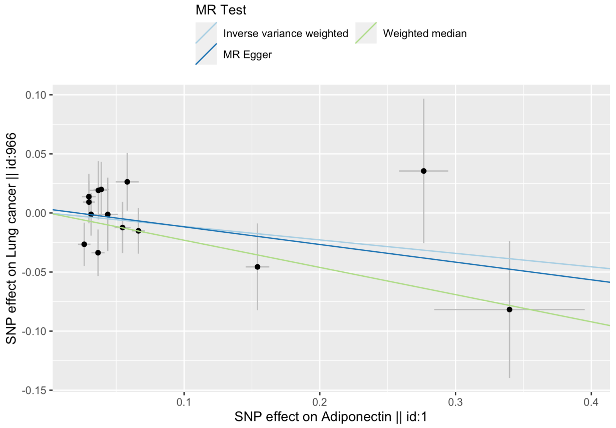

Circulating adiponectin

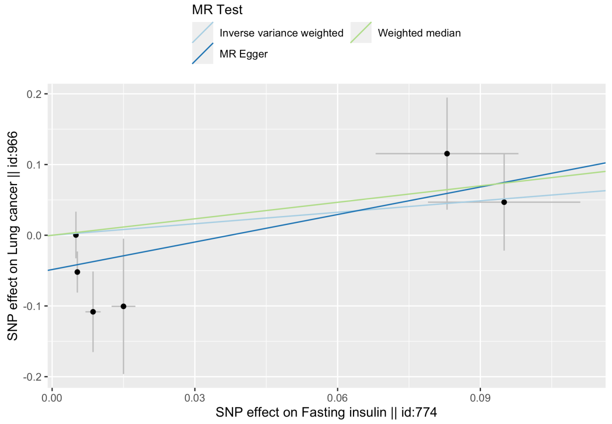

Fasting insulin interaction with body mass index

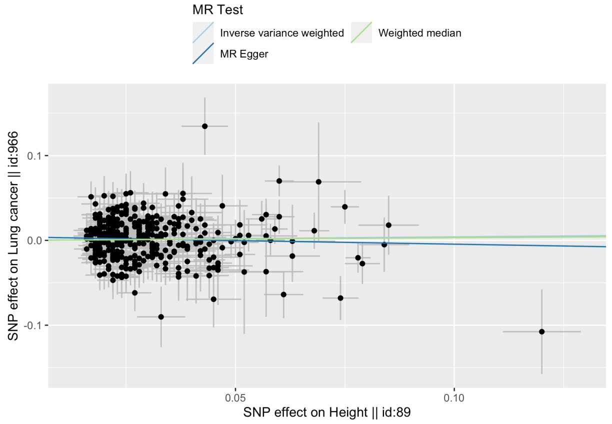

Adult height

7/7

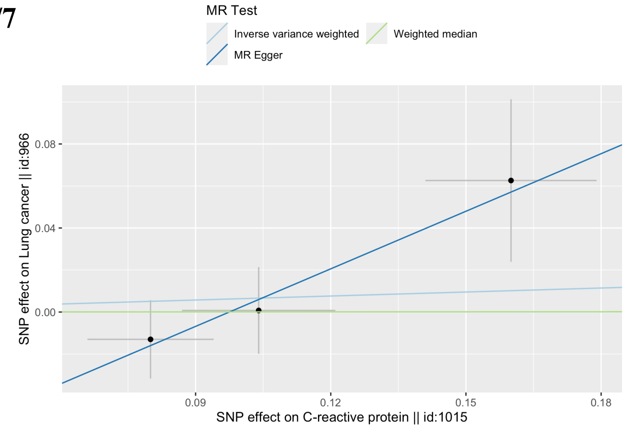

Serum C-reactive protein

Supplementary figure 3. Funnel plots for the assessment of directional pleiotropy in the MR analysis between putative modifiable risk factors and lung cancer.

1/7

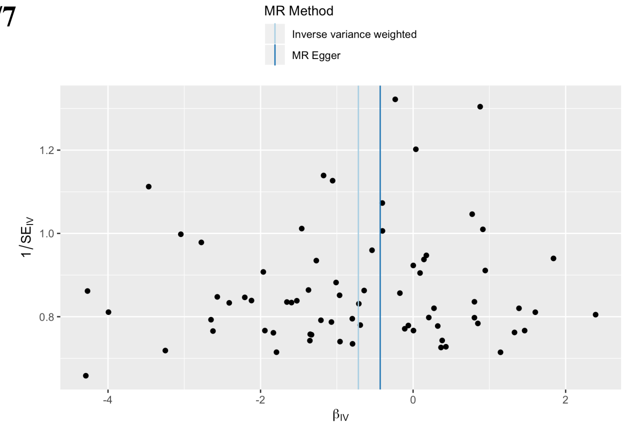

Years of schooling

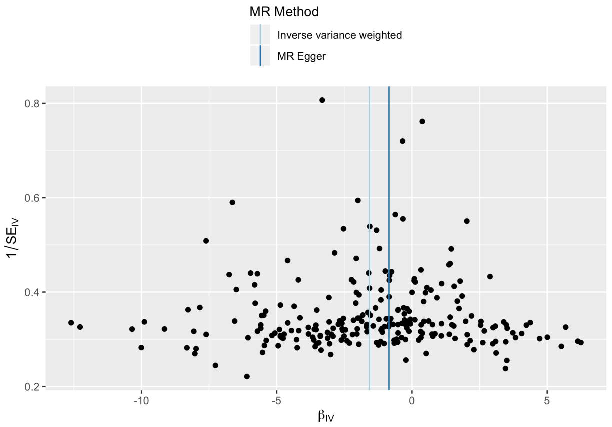

College or university degree

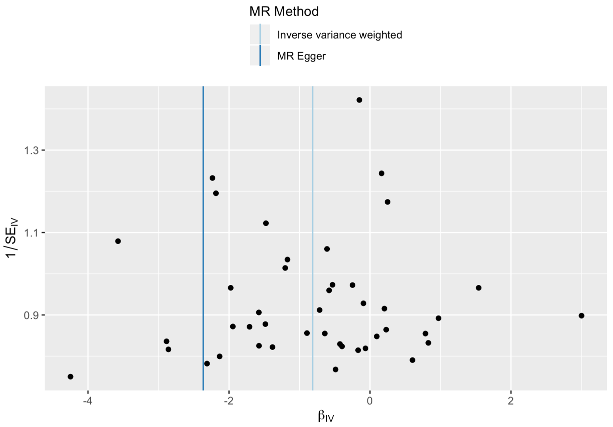

Household income

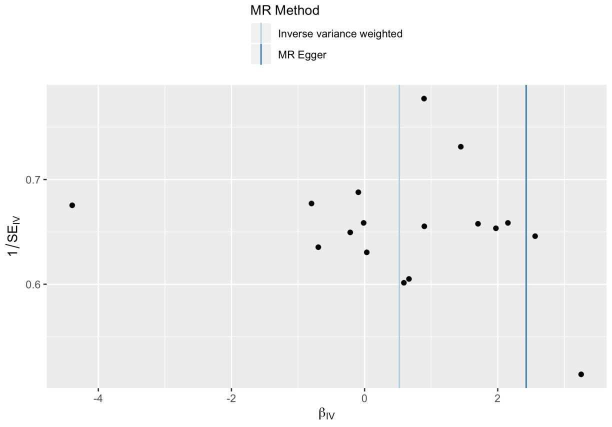

Townsend deprivation index

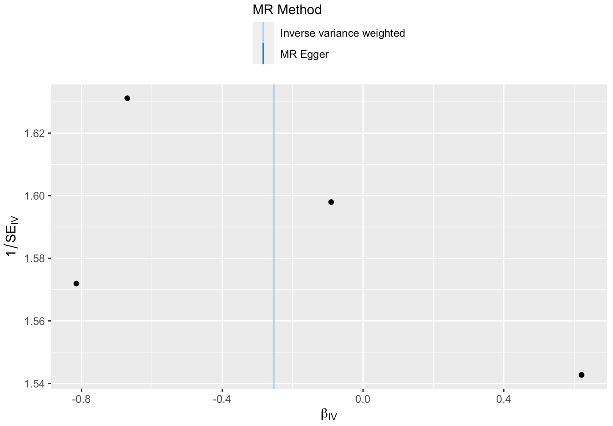

Accelerometer-based physical activity

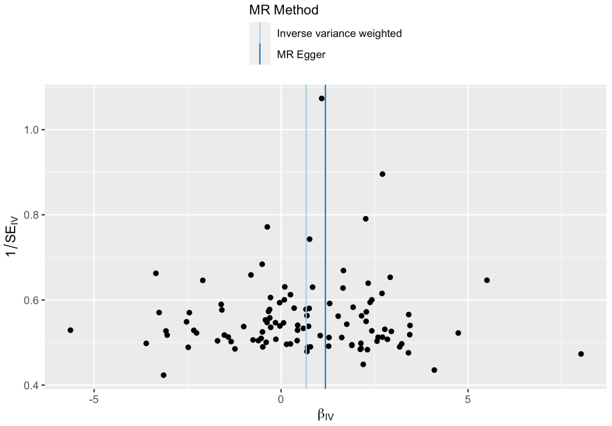

Time spent watching television

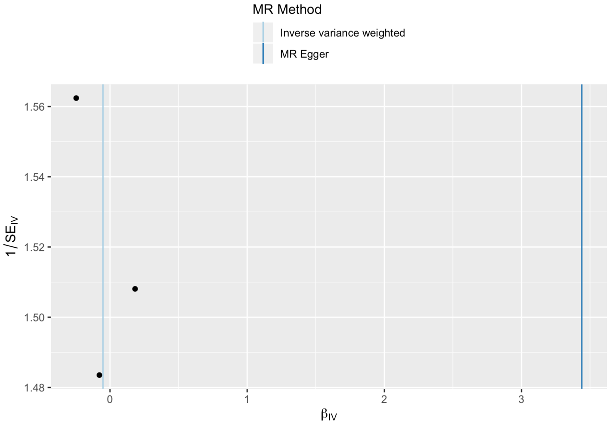

Sedentary behaviours

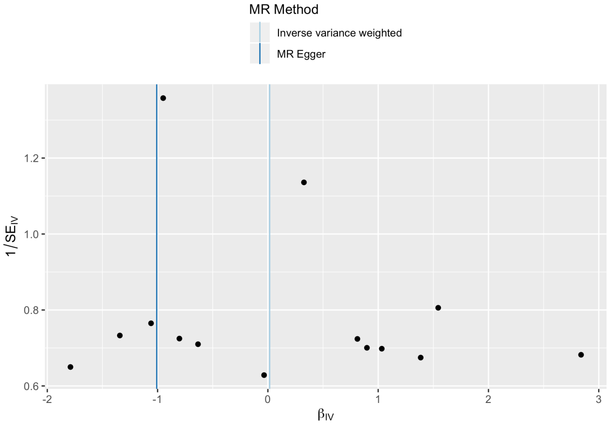

Bowls of cereal per week

2/7

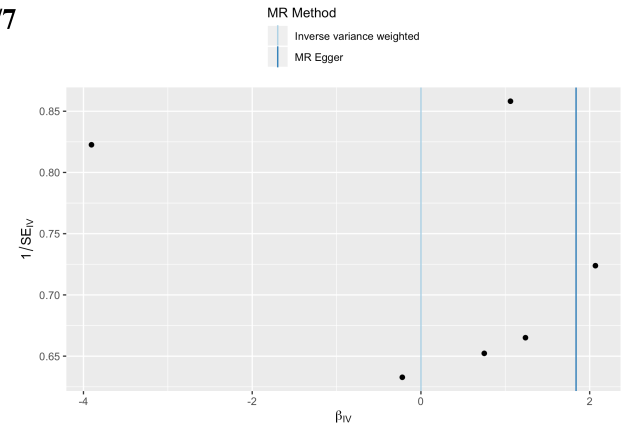

Tablespoons of cooked vegetables per day

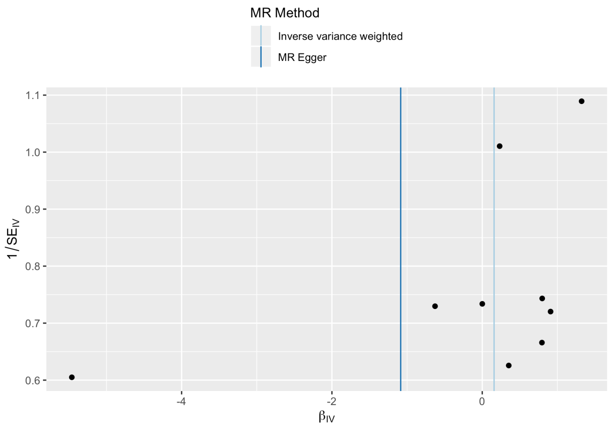

Tablespoons of raw vegetables per day

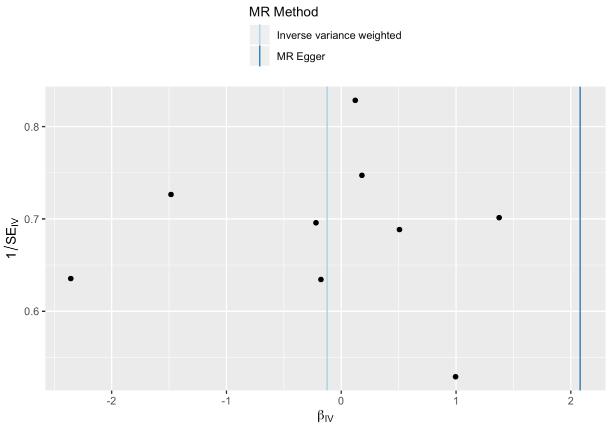

Pieces of dried fruit per day

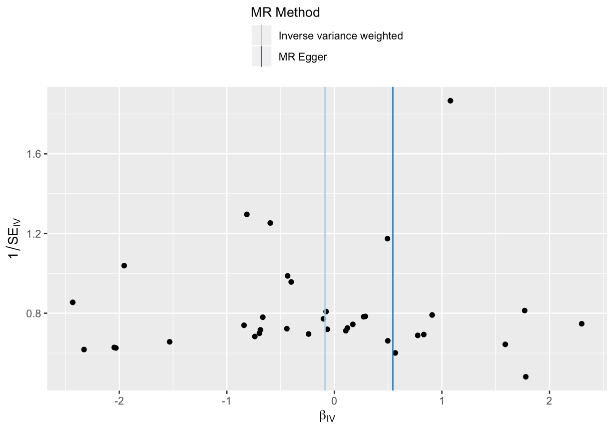

Pieces of fresh fruit per day

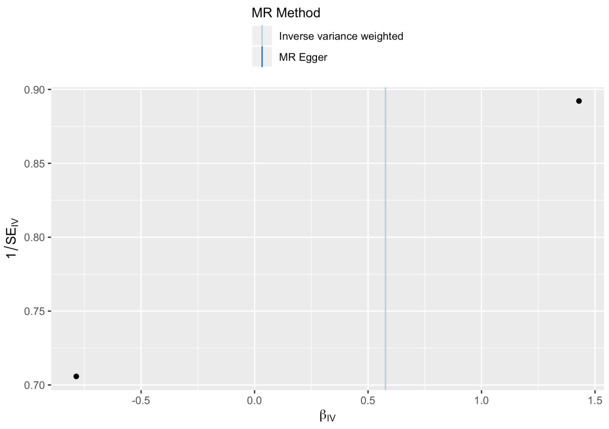

Overall beef intake

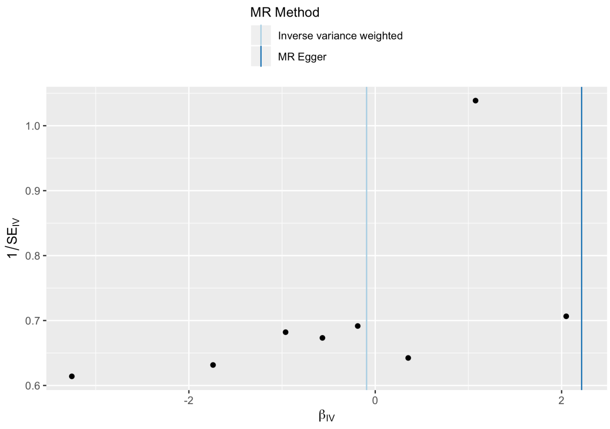

Overall lamb/mutton intake

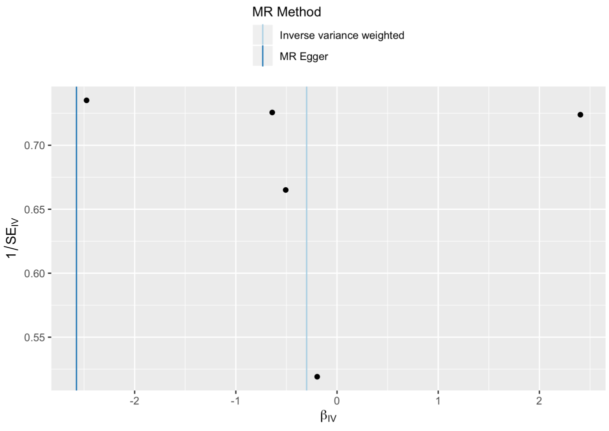

Overall pork intake

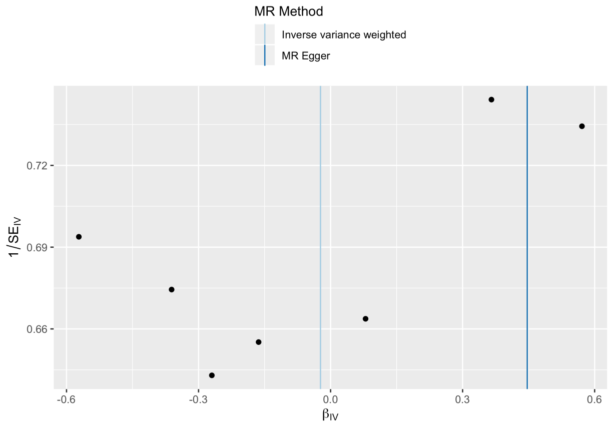

Processed meat intake

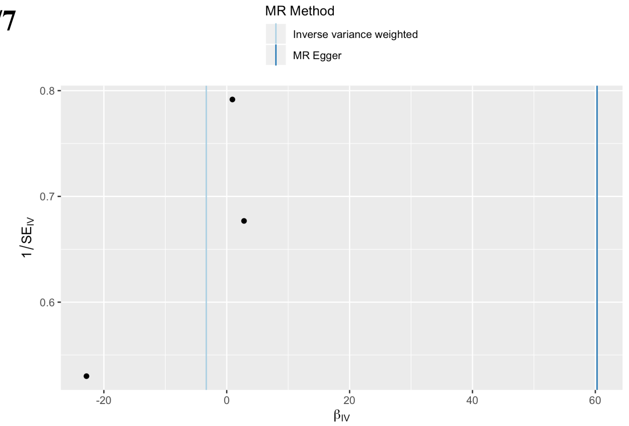

Poultry intake

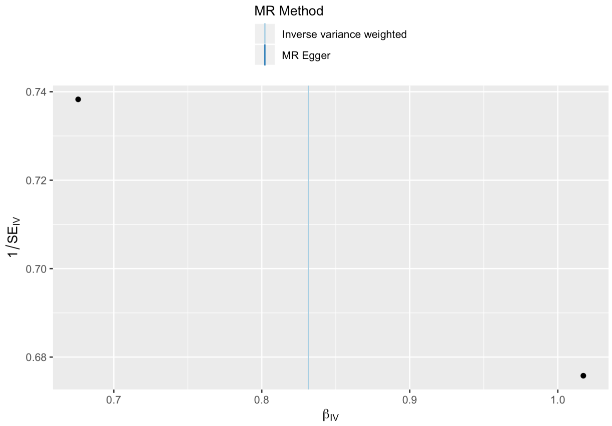

Overall non-oily fish intake

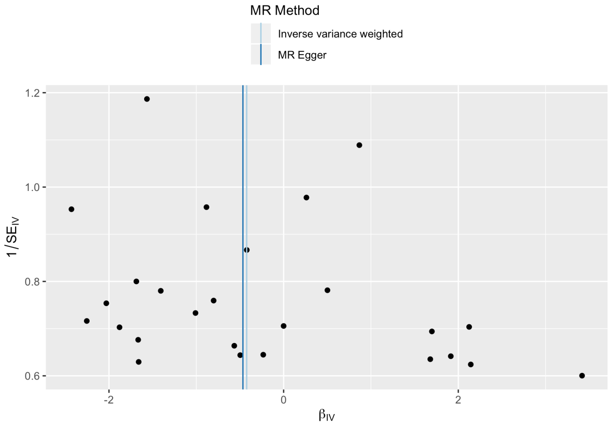

Overall oily fish intake

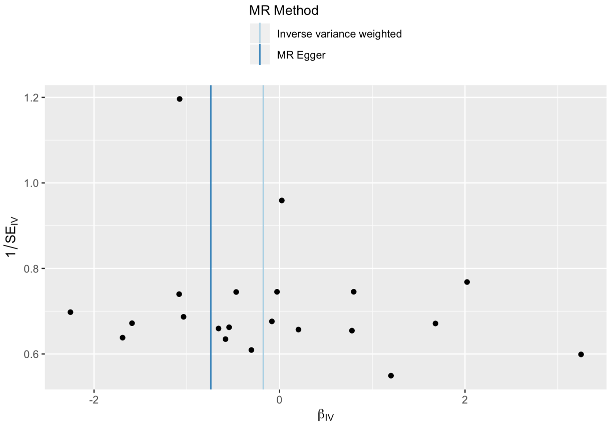

Overall alcohol intake

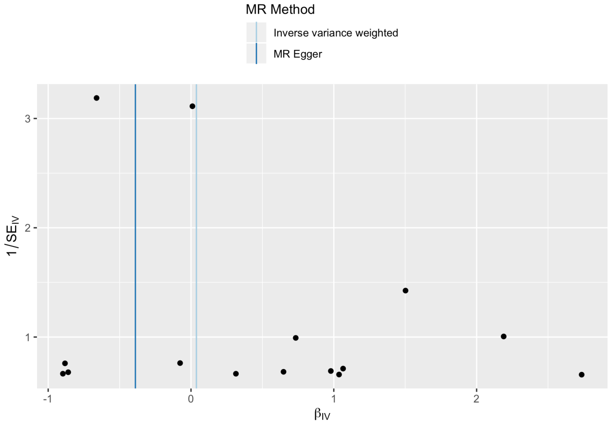

Cups of coffee per day

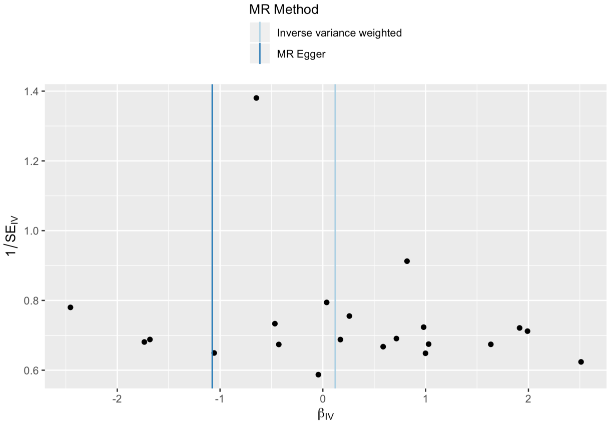

Cups of tea per day

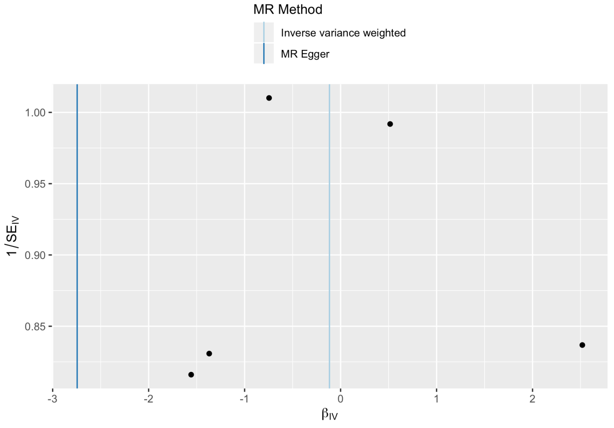

Carbohydrate intake

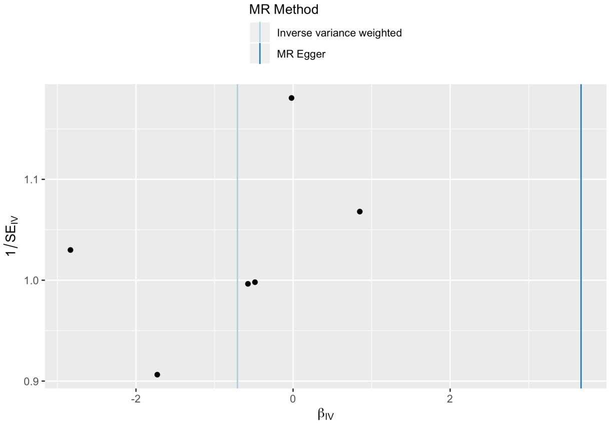

Protein intake

4/7

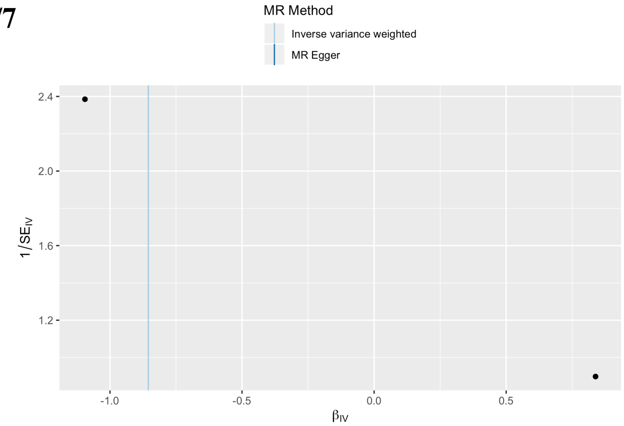

Fat intake

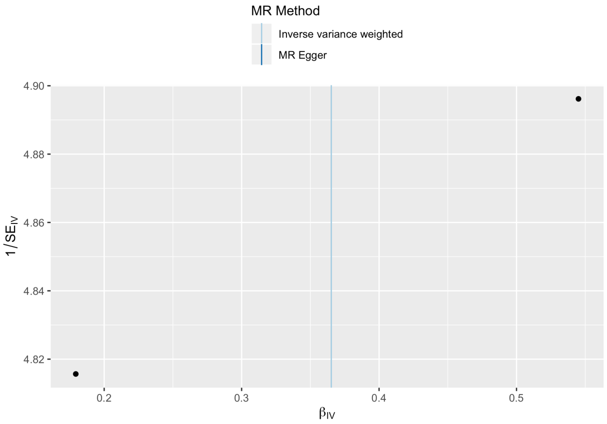

Serum vitamin A1 (Retinol)

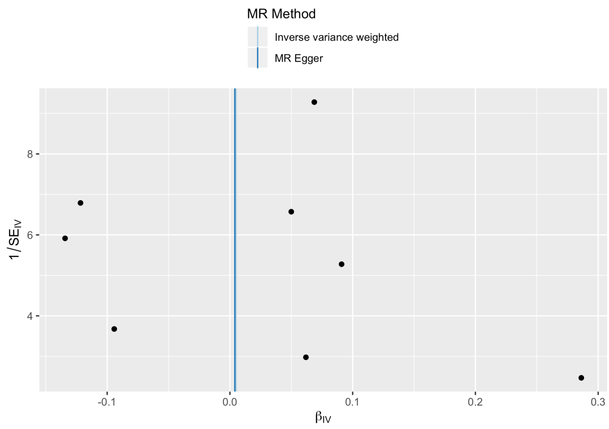

Serum vitamin B12

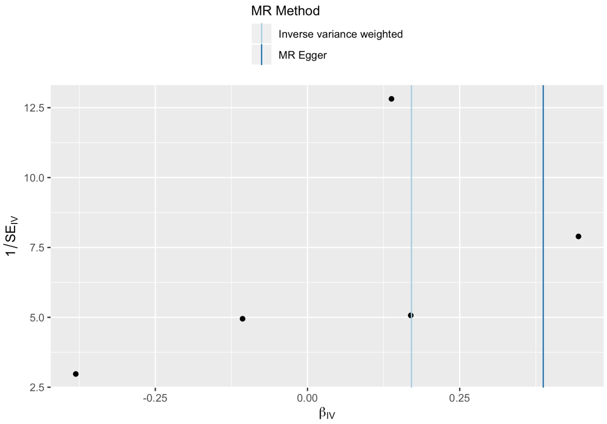

Circulating hydroxyvitamin D

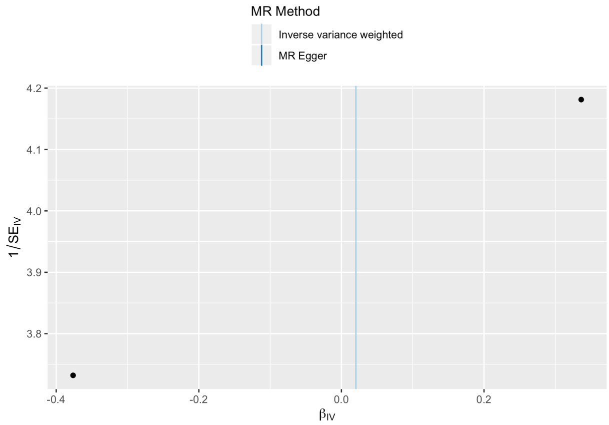

Serum vitamin E

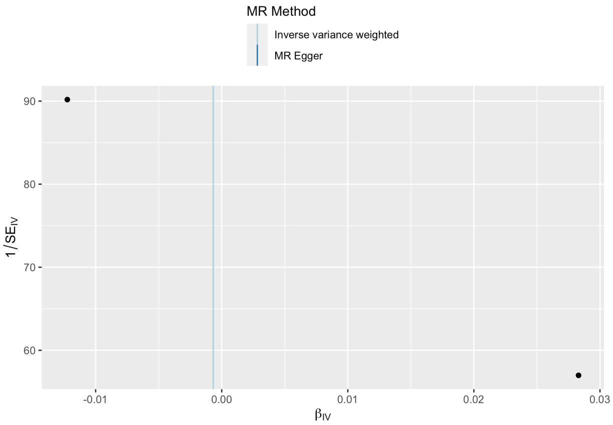

Inorganic arsenic in urine (%) (iAs%)

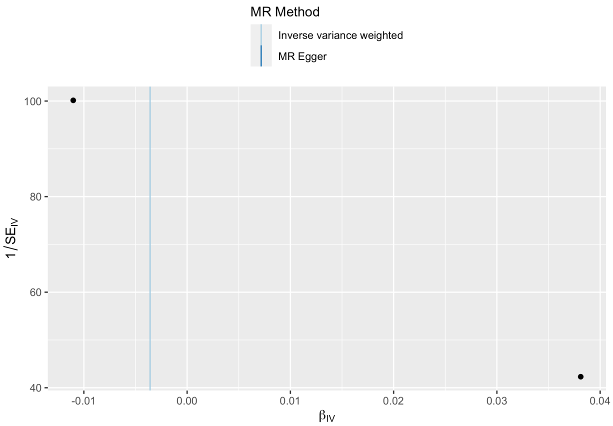

Monomethylarsenate in urine (%) (MMA%)

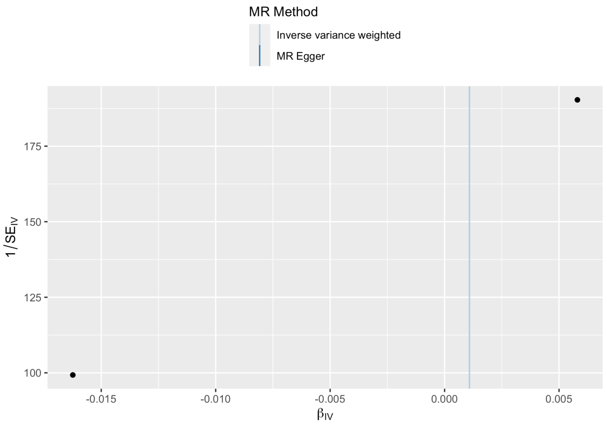

Dimethylarsinate in urine (%) (DMA%)

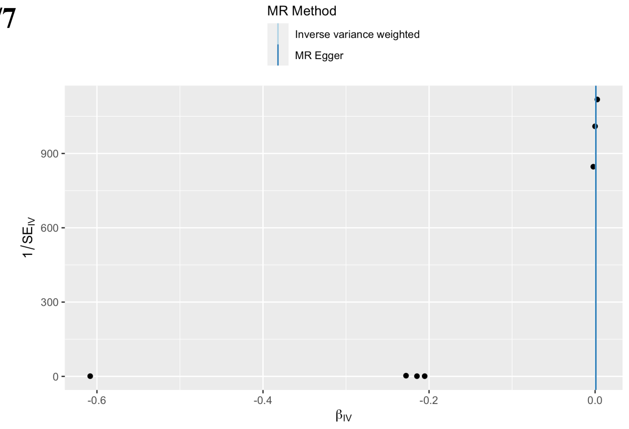

Serum calcium

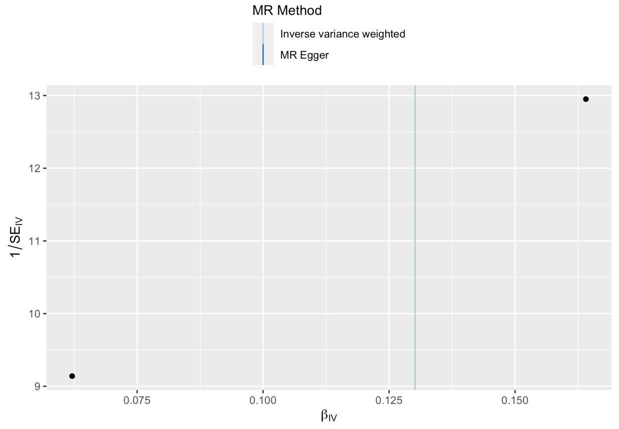

Cooper in blood

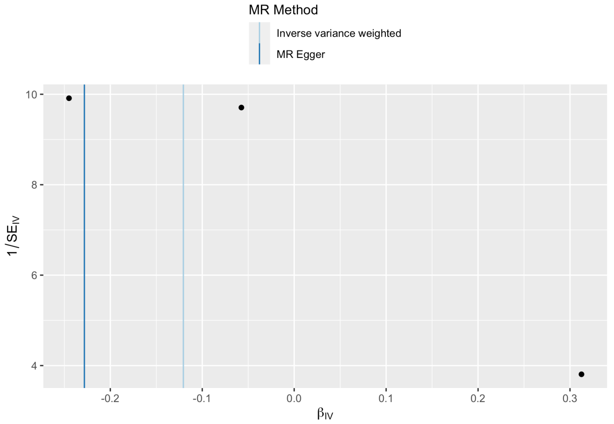

Biochemical markers for iron status

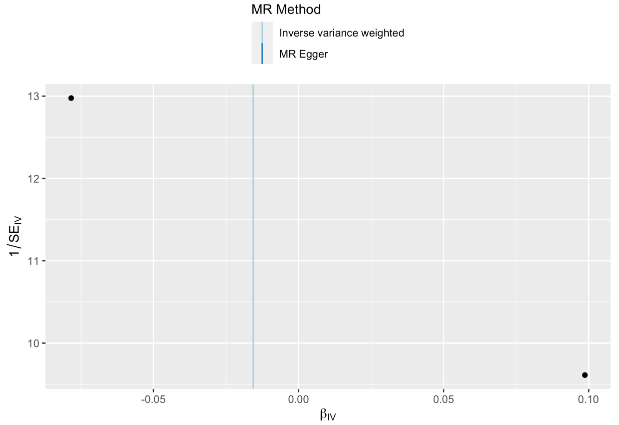

Zinc in blood

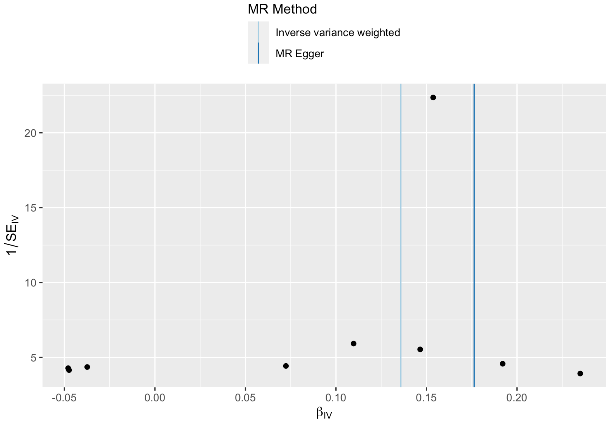

Other polyunsaturated fatty acids than 18:2 in blood

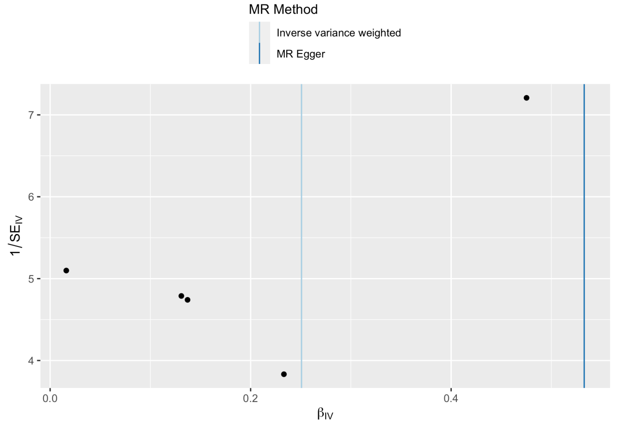

Docosahexaenoic acid (DHA) (22:6n-3) in blood

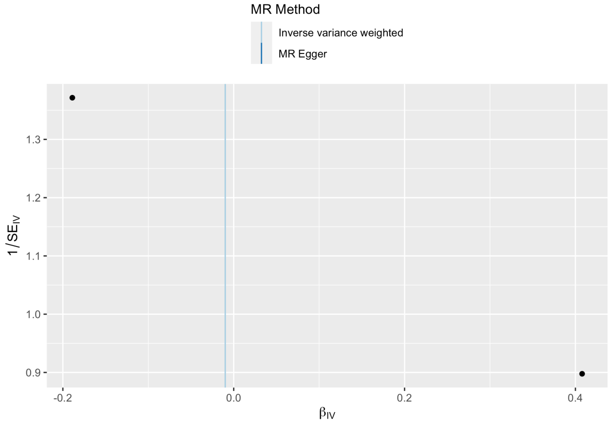

Dihomo-γ-linolenic acid (DGLA) (20:3n-6) in blood

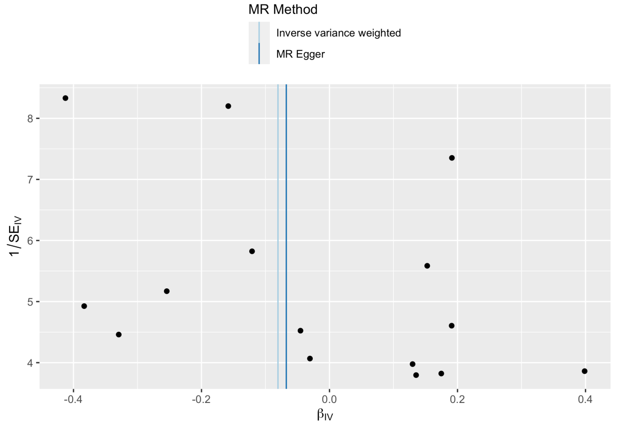

Linoleic acid (LA) (18:2n-6) in blood

6/7

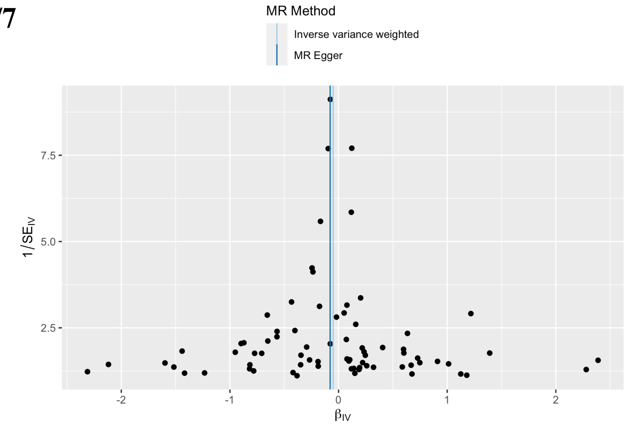

Low-density lipoprotein cholesterol level in blood

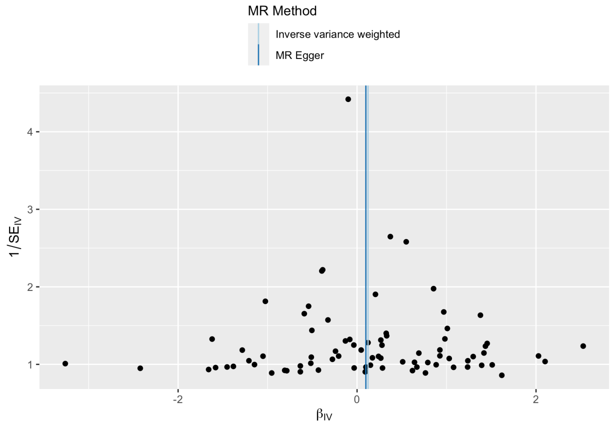

Body mass index

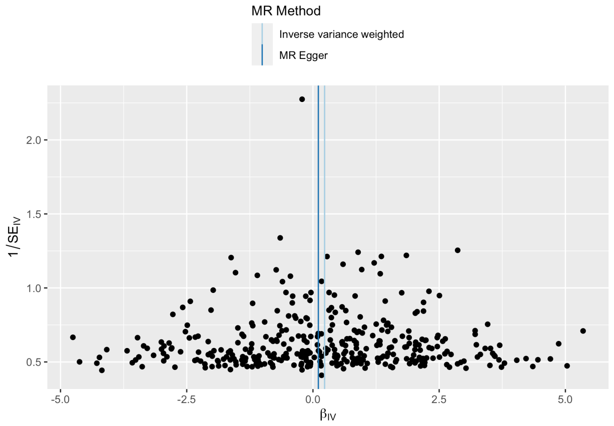

Body fat percentage

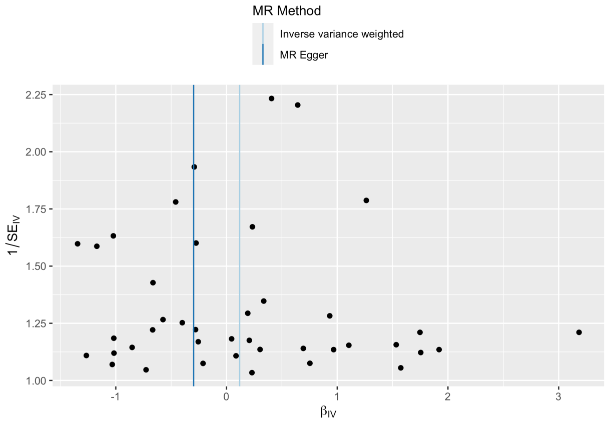

Waist circumference

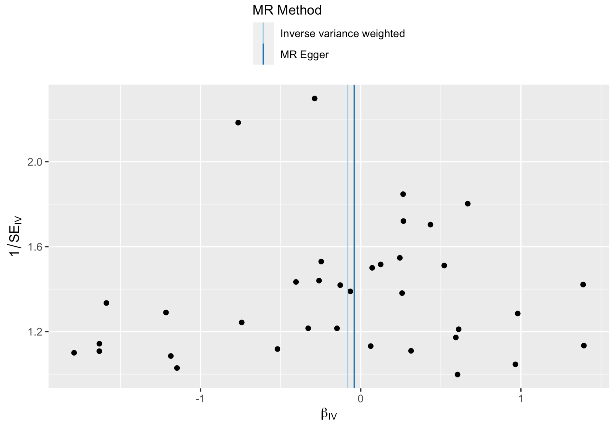

Waist to hip ratio

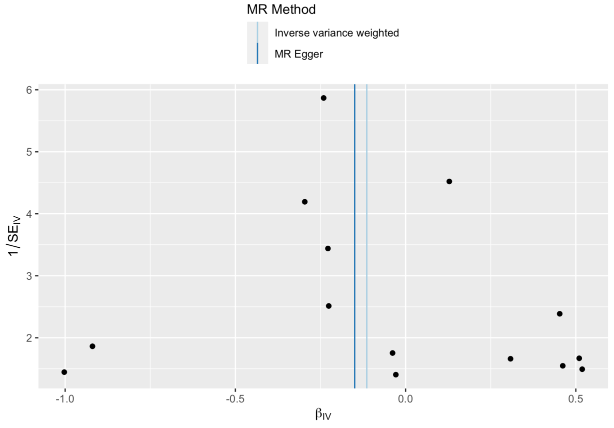

Circulating adiponectin

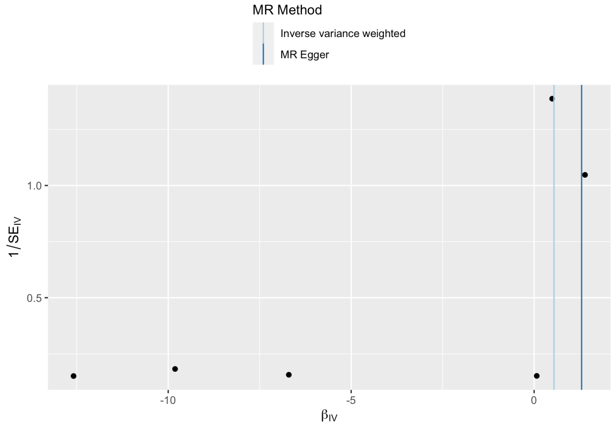

Fasting insulin interaction with body mass index

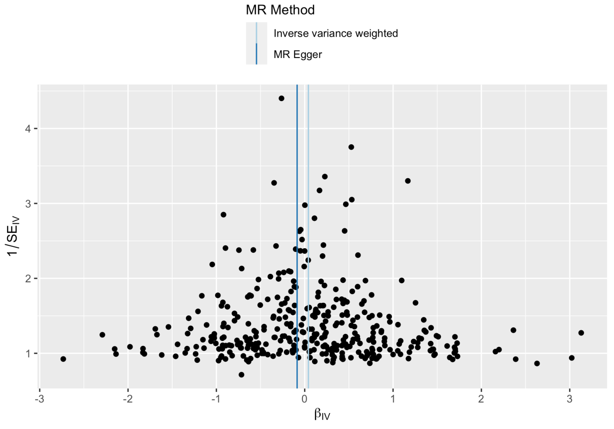

Adult height

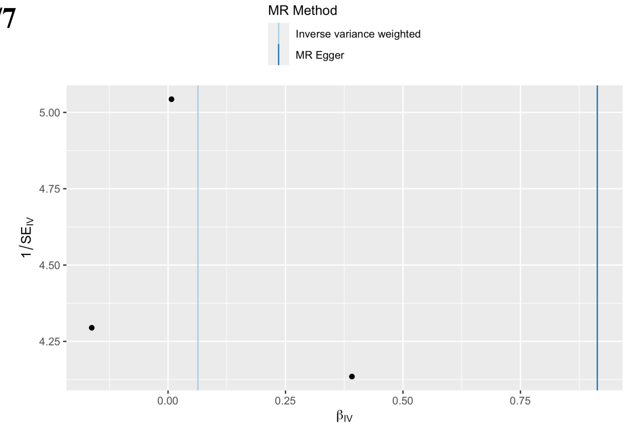

Serum C-reactive protein

Supplementary figure 4. MR estimates [presented as log_{10}(odds ratio)] of the relationship between the putative modifiable risk factors and lung adenocarcinoma.

a) Significant risk factors of lung cancer; b) Suggestive risk factors of lung cancer; c) The line of the forest plot for this variable was not shown because its odds ratio was too large. d) MR estimate of this variable was derived from MR Egger, for the adjustment of the detected directional pleiotropy.

Potential modifiable risk factor	OR (95% CI)	P-value
**Socioeconomic**		
Years of schooling	0.64 (0.41 - 1.02)	6.02E-02
College or university degree	0.26 (0.15 - 0.44) [a]	6.45E-07
Unemployed	0.00 (0.00 - 275.46) [c]	2.01E-01
In paid employment or self-employed	722.74 (0.04 - 12695027.60) [c]	1.87E-01
Household income	0.03 (0.00 - 0.25) [b, d]	2.65E-03
Townsend deprivation index	2.29 (0.76 - 6.93)	1.41E-01
**Lifestyle**		
Cigarettes smoked per day	1.32 (1.23 - 1.42) [a]	1.24E-14
Accelerometer-based physical activity	0.52 (0.20 - 1.33)	1.71E-01
Time spent watching television	1.81 (1.02 - 3.21) [b]	4.14E-02
Sedentary behaviours	0.52 (0.17 - 1.63)	2.61E-01
**Dietary**		
Bowls of cereal per week	1.78 (0.66 - 4.76)	2.53E-01
Tablespoons of cooked vegetables per day	0.40 (0.04 - 3.70)	4.17E-01
Tablespoons of raw vegetables per day	2.58 (0.73 - 9.07)	1.40E-01
Pieces of dried fruit per day	2.21 (0.51 - 9.56)	2.87E-01
Pieces of fresh fruit per day	0.79 (0.44 - 1.43)	4.38E-01
Overall beef intake	0.88 (0.06 - 12.81)	9.27E-01
Overall lamb/mutton intake	0.48 (0.11 - 2.11)	3.33E-01
Overall pork intake	3.00 (0.31 - 29.57)	3.46E-01
Processed meat intake	0.42 (0.08 - 2.16)	2.97E-01
Poultry intake	0.01 (0.00 - 2171.26) [c]	4.45E-01
Overall non-oily fish intake	2.67 (0.13 - 56.36)	5.29E-01
Overall oily fish intake	0.55 (0.26 - 1.16)	1.17E-01
Never eat eggs vs. no eggs restrictions	6.59 (0.00 - 104147.59) [c]	7.02E-01
Overall alcohol intake	0.73 (0.30 - 1.77)	4.90E-01
Cups of coffee per day	0.95 (0.54 - 1.67)	8.61E-01
Cups of tea per day	1.11 (0.46 - 2.66)	8.24E-01
Carbohydrate intake	2.00 (0.45 - 8.80)	3.62E-01
Protein intake	0.45 (0.12 - 1.74)	2.46E-01
Fat intake	0.20 (0.06 - 0.72) [b]	1.35E-02
Serum vitamin A1 (Retinol)	1.49 (0.96 - 2.32)	7.37E-02
Vitamin B6 blood concentration	1.01 (0.97 - 1.06)	5.44E-01
Serum vitamin B12	1.24 (1.04 - 1.49) [b]	1.88E-02
Circulating hydroxyvitamin D	1.22 (0.94 - 1.57)	1.33E-01
Serum vitamin E	1.24 (0.72 - 2.12)	4.35E-01
Homocysteine blood concentration	1.02 (0.98 - 1.07)	3.13E-01
Circulating carotenoids	0.86 (0.68 - 1.08)	1.98E-01
Inorganic arsenic in urine (%) (iAs%)	1.01 (0.96 - 1.07)	6.40E-01
Monomethylarsenate in urine (%) (MMA%)	1.01 (0.95 - 1.07)	7.82E-01
Dimethylarsinate in urine (%) (DMA%)	0.99 (0.97 - 1.02)	7.13E-01
Serum calcium	1.00 (1.00 - 1.00)	5.22E-02
Cooper in blood	1.15 (0.95 - 1.39)	1.64E-01
Biochemical markers for iron status	0.91 (0.63 - 1.31)	6.14E-01
Selenium in blood	1.09 (0.86 - 1.39)	4.82E-01
Zinc in blood	0.99 (0.70 - 1.41)	9.72E-01
Other polyunsaturated fatty acids than 18:2 in blood	1.20 (1.07 - 1.35) [b]	1.57E-03
Docosahexaenoic acid (DHA) (22:6n-3) in blood	1.38 (1.03 - 1.85) [b]	3.23E-02
Docosapentaenoic acid (DPA) (22:5n-3) in blood	15.22 (4.33 - 53.58) [a]	2.22E-05
Eicosapentaenoic acid (EPA) (20:5n-3) in blood	12.94 (2.88 - 58.14) [b]	8.35E-04
Arachidonic acid (AA) (20:4n-6) in blood	6.66 (2.25 - 19.74) [a]	6.25E-04
Dihomo- -linolenic acid (DGLA) (20:3n-6) in blood	1.04 (0.17 - 6.49)	9.71E-01
Linoleic acid (LA) (18:2n-6) in blood	0.86 (0.71 - 1.04)	1.24E-01
Low-density lipoprotein cholesterol level in blood	1.00 (0.84 - 1.19)	9.97E-01
**Cardiometabolic**		
Body mass index	0.89 (0.70 - 1.14)	3.69E-01
Body fat percentage	1.10 (0.85 - 1.43)	4.60E-01
Waist circumference	0.85 (0.60 - 1.20)	3.49E-01
Waist to hip ratio	0.78 (0.51 - 1.20)	2.58E-01
Circulating adiponectin	0.65 (0.42 - 1.00) [d]	7.48E-02
Fasting insulin interaction with body mass index	4.93 (0.73 - 33.36)	1.02E-01
**Developmental and growth factors**		
Adult height	1.05 (0.93 - 1.19)	4.00E-01
**Inflammatory**		
Serum C-reactive protein	1.13 (0.73 - 1.76)	5.76E-01

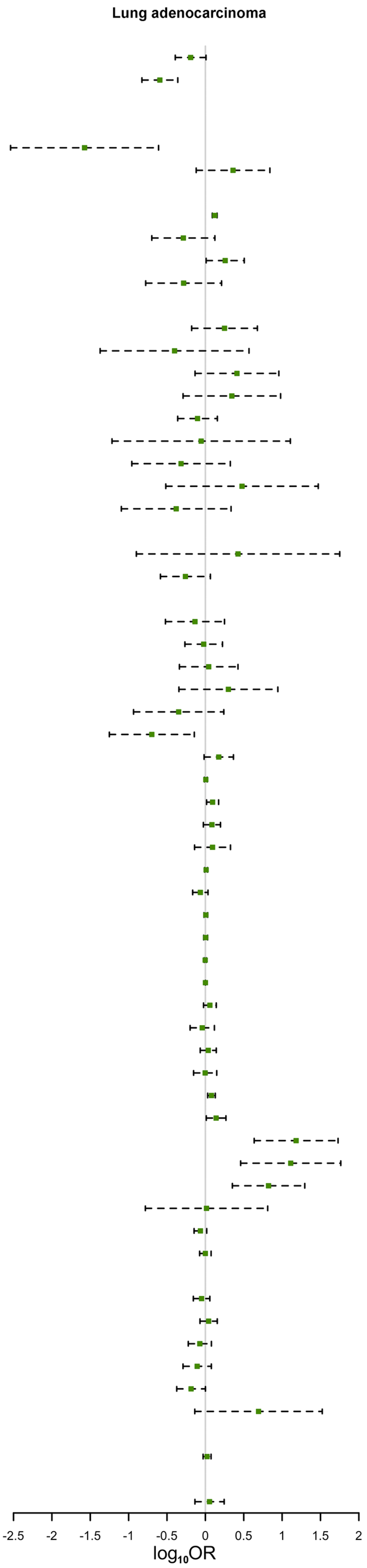

Supplementary figure 5. MR estimates [presented as $\log_{10}$(odds ratio)] of the relationship between the putative modifiable risk factors and lung squamous cell carcinoma.

a) Significant risk factors of lung cancer; b) Suggestive risk factors of lung cancer; c) The line of the forest plot for this variable was not shown because its odds ratio was too large.

Potential modifiable risk factor	Lung squamous cell carcinoma	OR (95% CI)	P-value
**Socioeconomic**			
Years of schooling		0.40 (0.26 - 0.62) [a]	2.93E-05
College or university degree		0.14 (0.08 - 0.25) [a]	4.52E-11
Unemployed		0.43 (0.00 - 19317698.40) [c]	9.25E-01
In paid employment or self-employed		3.26 (0.00 - 134262.68) [c]	8.28E-01
Household income		0.41 (0.24 - 0.68) [a]	6.33E-04
Townsend deprivation index		1.23 (0.40 - 3.75)	7.19E-01
**Lifestyle**			
Cigarettes smoked per day		1.34 (1.25 - 1.44) [a]	2.06E-15
Accelerometer-based physical activity		0.75 (0.29 - 1.94)	5.48E-01
Time spent watching television		3.03 (1.71 - 5.35) [a]	1.40E-04
Sedentary behaviours		1.02 (0.32 - 3.23)	9.69E-01
**Dietary**			
Bowls of cereal per week		0.67 (0.19 - 2.38)	5.40E-01
Tablespoons of cooked vegetables per day		4.54 (0.45 - 45.76)	1.99E-01
Tablespoons of raw vegetables per day		0.55 (0.13 - 2.37)	4.26E-01
Pieces of dried fruit per day		0.56 (0.10 - 3.06)	5.05E-01
Pieces of fresh fruit per day		0.96 (0.53 - 1.73)	8.85E-01
Overall beef intake		1.52 (0.04 - 56.85)	8.21E-01
Overall lamb/mutton intake		0.99 (0.23 - 4.30)	9.94E-01
Overall pork intake		0.29 (0.02 - 3.50)	3.31E-01
Processed meat intake		0.96 (0.19 - 4.99)	9.65E-01
Poultry intake		0.13 (0.00 - 149177.70) [c]	7.73E-01
Overall non-oily fish intake		1.17 (0.06 - 22.67)	9.17E-01
Overall oily fish intake		0.67 (0.25 - 1.77)	4.20E-01
Never eat eggs vs. no eggs restrictions		9010.43 (0.11 - 708289167.00) [c]	1.13E-01
Overall alcohol intake		0.60 (0.23 - 1.55)	2.88E-01
Cups of coffee per day		1.18 (0.53 - 2.61)	6.89E-01
Cups of tea per day		1.04 (0.37 - 2.95)	9.38E-01
Carbohydrate intake		0.53 (0.04 - 7.77)	6.46E-01
Protein intake		0.65 (0.15 - 2.73)	5.56E-01
Fat intake		0.38 (0.11 - 1.31)	1.26E-01
Serum vitamin A1 (Retinol)		1.06 (0.40 - 2.78)	9.11E-01
Vitamin B6 blood concentration		0.98 (0.94 - 1.02)	3.34E-01
Serum vitamin B12		0.86 (0.72 - 1.04)	1.16E-01
Circulating hydroxyvitamin D		1.06 (0.89 - 1.28)	5.11E-01
Serum vitamin E		1.08 (0.27 - 4.39)	9.14E-01
Homocysteine blood concentration		1.02 (0.98 - 1.07)	3.87E-01
Circulating carotenoids		1.23 (0.99 - 1.52)	5.78E-02
Inorganic arsenic in urine (%) (iAs%)		0.99 (0.90 - 1.09)	7.78E-01
Monomethylarsenate in urine (%) (MMA%)		0.98 (0.90 - 1.07)	6.39E-01
Dimethylarsinate in urine (%) (DMA%)		1.01 (0.96 - 1.06)	7.09E-01
Serum calcium		1.00 (1.00 - 1.00)	8.17E-01
Cooper in blood		1.05 (0.82 - 1.35)	6.78E-01
Biochemical markers for iron status		0.75 (0.61 - 0.91) [b]	4.59E-03
Selenium in blood		0.91 (0.72 - 1.14)	4.15E-01
Zinc in blood		1.02 (0.85 - 1.23)	8.19E-01
Other polyunsaturated fatty acids than 18:2 in blood		1.16 (1.03 - 1.30) [b]	1.44E-02
Docosahexaenoic acid (DHA) (22:6n-3) in blood		1.19 (0.85 - 1.67)	3.06E-01
Docosapentaenoic acid (DPA) (22:5n-3) in blood		8.97 (2.59 - 31.01) [a]	5.28E-04
Eicosapentaenoic acid (EPA) (20:5n-3) in blood		11.33 (2.52 - 50.97) [b]	1.55E-03
Arachidonic acid (AA) (20:4n-6) in blood		5.41 (1.81 - 16.18) [b]	2.54E-03
Dihomo- -linolenic acid (DGLA) (20:3n-6) in blood		0.53 (0.08 - 3.57)	5.12E-01
Linoleic acid (LA) (18:2n-6) in blood		0.90 (0.76 - 1.05)	1.74E-01
Low-density lipoprotein cholesterol level in blood		0.96 (0.83 - 1.12)	6.30E-01
**Cardiometabolic**			
Body mass index		1.37 (1.02 - 1.85) [b]	3.92E-02
Body fat percentage		1.32 (1.02 - 1.72) [b]	3.45E-02
Waist circumference		1.19 (0.72 - 1.97)	4.96E-01
Waist to hip ratio		1.07 (0.75 - 1.52)	7.21E-01
Circulating adiponectin		0.76 (0.56 - 1.04)	8.55E-02
Fasting insulin interaction with body mass index		2.89 (0.14 - 58.62)	4.90E-01
**Developmental and growth factors**			
Adult height		1.00 (0.90 - 1.11)	9.73E-01
**Inflammatory**			
Serum C-reactive protein		1.26 (0.86 - 1.85)	2.29E-01

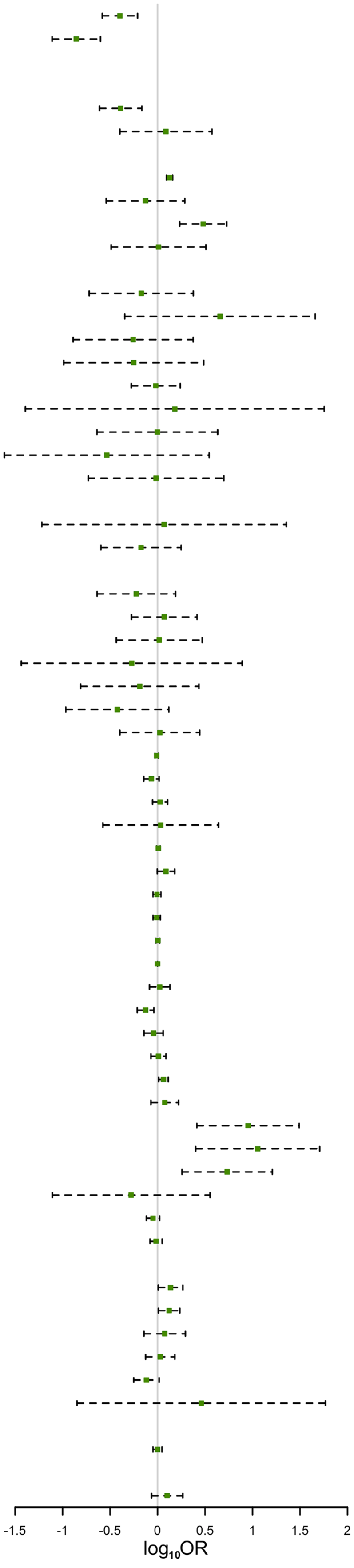

Supplement: Supplementary file 1 — Fig S1‐5 [file CAM4-10-4587-s001.pdf]
